# Changes in soil taxonomic and functional diversity resulting from gamma irradiation

**DOI:** 10.1038/s41598-019-44441-7

**Published:** 2019-05-27

**Authors:** Matthew Chidozie Ogwu, Dorsaf Kerfahi, HoKyung Song, Ke Dong, Hoseong Seo, Sangyong Lim, Sathiyaraj Srinivasan, Myung Kyum Kim, Bruce Waldman, Jonathan M. Adams

**Affiliations:** 1 School of Biological Sciences, Seoul National University, 1 Gwanak-ro, Gwanak-gu, Seoul, 08826 Republic of Korea; 2 Department of Plant Biology and Biotechnology, University of Benin, PMB 1154, Ugbowo, Benin City, Edo State Nigeria; 3 Department of Life Sciences, Kyonggi University, Suwon, 443-760 Republic of Korea; 4 Korea Atomic Energy Research Institute, 111 Daedeok-Daero, 989 Beon-Gil, Yuseong-gu, Daejeon, Republic of Korea; 5 Department of Bio and Environmental Technology, Division of Environmental and Life Science, College of Natural Science, Seoul Women’s University, 623 Hwarangno, Nowon-gu, Seoul, 139-774 Republic of Korea; 6 Department of Integrative Biology, Oklahoma State University, 501 Life Sciences West, Stillwater, Oklahoma 74078 USA; 7 School of Geographic and Oceanographic Sciences, Nanjing University, Nanjing 210023, Qixia District, Jiangsu Province People’s Republic of China

**Keywords:** Microbial ecology, Ecosystem ecology

## Abstract

Little is known of the effects of ionizing radiation exposure on soil biota. We exposed soil microcosms to weekly bursts of ^60^Co gamma radiation over six weeks, at three levels of exposure (0.1 kGy/hr/wk [low], 1 kGy/hr/wk [medium] and 3 kGy/hr/wk [high]). Soil DNA was extracted, and shotgun metagenomes were sequenced and characterised using MG-RAST. We hypothesized that with increasing radiation exposure there would be a decrease in both taxonomic and functional diversity. While bacterial diversity decreased, diversity of fungi and algae unexpectedly increased, perhaps because of release from competition. Despite the decrease in diversity of bacteria and of biota overall, functional gene diversity of algae, bacteria, fungi and total biota increased. Cycles of radiation exposure may increase the range of gene functional strategies viable in soil, a novel ecological example of the effects of stressors or disturbance events promoting some aspects of diversity. Moreover, repeated density-independent population crashes followed by population expansion may allow lottery effects, promoting coexistence. Radiation exposure produced large overall changes in community composition. Our study suggests several potential novel radiation-tolerant groups: in addition to Deinococcus-Thermus, which reached up to 20% relative abundance in the metagenome, the phyla Chloroflexi (bacteria), Chytridiomycota (fungi) and Nanoarcheota (archaea) may be considered as radiation-tolerant.

## Introduction

The effects of ionizing radiation on humans and the ecosystems have been investigated since the 1950s^1–7^ driven by interest in understanding radiation effects on both natural and agricultural ecosystems. Radiation effects are also of interest for exploring the viability of ecosystems in space travel and space colonization^8,9^, and in assessing the likelihood of independently evolving biota in high radiation environments on other planets^10,11^.

Radiation is known to affect living cells in diverse ways. There are direct effects on the structural integrity of cellular components, such as membranes^12–14^, and on enzyme viability by protein denaturation^15,16^. Cells are generally most vulnerable when actively dividing, due to the breakage or alteration of DNA that results from radiation^17^. Certain types of organisms are far more susceptible than others to radiation^18^; although some types of bacteria and archaea can survive at thousands of times the dosages that would kill humans^19^. The biological mechanism of radiation resistance differs between taxa and involves the coordination of a myriad of biochemical and genetic processes. In the most radiation resistant groups known (*Deinococcus spp*. and *Thermococcus gammatolerans*), resistance involves genes for a complex network of DNA repair and metabolic switching processes^20,21^. Various other radiation resistance and repair mechanisms have also been identified in living organisms^22^.

However, the effects of high doses of ionizing radiation on complex communities of soil organisms have not been well studied. Soil is arguably the most biologically diverse environment on Earth^23^, and most of its diversity consists of complex communities of organisms that are microscopic or near microscopic, and morphologically cryptic^24^. Most of these microscopic life forms have neither been isolated nor studied^25,26^, yet these microorganisms play key roles in the ecosystem^27,28^. More thorough assessment of soil biological diversity, including the huge numbers of non-cultured forms, is possible due to advances in sequencing and bioinformatics. Using environmental DNA-based methods to study irradiated soils has the potential to uncover the full range of radiation-resistant forms of every group of organisms (be they bacteria, fungi, metazoa, protists, archaea or viruses).

The study of irradiated soil communities also may be approached from a generalized ecological point of view, as a system subject to stress and disturbance effects and recovery. This approach has the potential to provide clues to the processes that govern the assembly of communities in nature, including the mechanisms behind diversity and coexistence. In this study, we took this approach, focussing on a number of hypotheses regarding the effects on radiation on soil communities and their functional characteristics:

### Soils subjected to gamma irradiation will have a taxonomically distinct biota from control soils, and the diversity of this biota will differ according to the radiation dose received

It is expected that exposure to radiation will lead to lower taxonomic diversity, because high radiation is a universal stressor to which microbial communities have low overall adaptation and tolerance^29^. Survival at high doses of radiation appears to involve elaborate mechanisms that probably evolved in response to natural exposure to UV radiation, drying and extreme heat^22,30,31^. It is likely that only a subset of taxa may happen to carry the adaptive traits to survive a large dose of radiation, which will result in a progressive diminution of diversity. Furthermore, we hypothesized a large shift in taxonomic composition of the soil biota, in terms of both lower and higher-level taxa, as some taxa will happen to possess stronger adaptations for radiation tolerance than others do. We hypothesized that at the highest level, Bacteria and Archaea would become relatively more abundant than Eukaryota, and especially Metazoa, due to the simpler cellular organization of these organisms^32^.

### Soils exposed to radiation will have a lower diversity of functional genes owing to the reduced diversity of taxa that can survive, and the restrictions imposed by radiation damage to physiological/ecological strategies

We anticipated that the extreme physiological and biochemical challenges presented by repeated radiation exposure would result in a reduced range of functional genes comprising the metagenome. Partly, this would be an incidental effect of reduced taxonomic diversity – reducing the range of genes particular to individual taxa. However, we also hypothesized that the damage caused by high radiation would make a range of energy-requiring physiological or biochemical functions non-viable^33^, because so much of the energy and resources of the cell would be diverted into radiation damage protection and repair mechanisms, thereby restricting the potential range of niches and ecological strategies.

### Greater abundance of certain key groups of genes will be associated with radiation exposure

These would include stress-response genes, which assist in the survival of various extreme conditions such as high temperatures, freezing or high salinity through forming biofilms, fruiting bodies, filaments, spores or taxis responses – often through regulating other downstream genes^34,35^. We envisaged that this occurs mainly through pre-adaptation after having evolved in the organisms to cope with damage caused by other stressors, and organisms carrying such genes are then ecologically selected, becoming more abundant in soil exposed to radiation.

Dormancy and sporulation-related genes were also hypothesized to become more abundant, because actively dividing cells are known to be particularly susceptible to radiation damage^36^. However, resistant species have the capacity to outlive it while others with the capacity to form resting spores or to enter periodic dormancy will be adapted to avoiding radiation damage^37,38^. Dormancy allows organisms to recover following disturbance in a way that other taxa cannot. Since radiation exposure in our experimental systems occurred in repeated bursts, rather than continuously, cells that were coincidentally dormant during exposure phases would be more likely to survive and then become abundant.

We also anticipated lower abundance of competition-related genes associated with radiation exposure. Each weekly radiation dose is likely to be associated with phases of mass death of cells, followed by a recovery phase in which surviving forms recolonize, exploiting the nutrients available from dead cells. This would be an example of an ‘r’ selected environment^39,40^, and such environments with abundant nutrients and space, are seen as ecologically selecting rapid growth and reproduction, rather than interference competition. Consequently, we expected to see a lower abundance of genes relating to antibiotic production (or antibiotic resistance to cope with this), and lower abundance of genes relating to cell-cell interactions (characterised as regulation and cell signalling, which includes programmed cell death and toxin-antitoxin systems related genes, proteolytic pathways related genes, quorum sensing and biofilm formation and regulation of virulence).

Additionally, as part of this study, we investigated basic soil parameters – total organic carbon, total nitrogen, and pH – to understand the extent to which the chemistry of the whole soil system may change either because of gamma (γ) ray exposure to the organisms^41^, or due to the effects of the radiation on soil chemistry. Chemical changes in soils that are completely sterilized by γ- rays are generally considered to be relatively minor^42–50^. However, γ-irradiation has been shown to induce surviving microbes to decompose soil organic matter, by influencing cellular metabolism and functionality without altering the soil structure^51,52^. We were interested in investigating the emergent properties of a changing soil system characterized by the survival of some living forms, resulting from repeated bursts of exposure. Whether these changes are just the purely chemical effects of repeated exposure to γ-rays, or consequences of shifts in soil biota community and activity might not be clear. Nevertheless, the possibility of such changes should be investigated as a part of the entire system, even if only as a spur to further study.

The radiation doses we used in this experiment were far higher than are seen at most radiation-contaminated sites: for example, a medium level nuclear waste site would be expected to experience around 20 Gy, while a high-level site or cooling pond might experience >1000 Gy^53^. Nevertheless, highly contaminated ecosystems after accidents have been recorded (100 Gy Chernobyl and 10 Gy at Fukushima^54,55^. Radiation doses in space would be around 0.5 mGy^56^. Even if doses as high as those we used are highly unusual on Earth, studying the extreme case of very high radiation doses may help in understanding the general direction in which selection from the lower radiation doses that are more commonly seen will tend to push the soil ecosystem. Cockell *et al*.^57^ has suggested that even normal background levels of radiation may be a significant long-term factor in soil systems.

## Results

### Soil chemical properties

**Figure 1:**
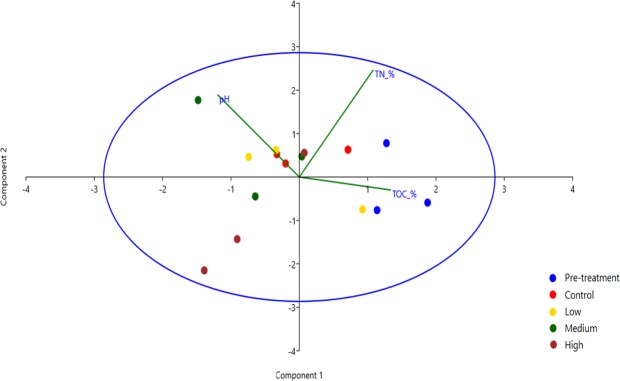
Principal component analysis of the effects of gamma irradiation on the measured environmental variables. Component 1 and 2 account for 83.60% of the observed variation. PCA loadings of component 1 suggest TN and TOC loaded positively on the axis while pH and TN loaded positively on the component 2.

Soil chemical properties differed among the treatment levels except for pH, which showed little variation across the treatments (Supplementary Fig. 1). The concentration of TN was highest in the pre-treatment samples and lowest in high radiation treated samples whereas TOC concentration, which was also highest in the pre-treatment, was lowest in the low radiation treated samples. TOC was significantly different between the treatment groups with P-_values_ (at p ≤ 0.05) (Supplementary Fig. 1). To understand the overall correlation effects, we conducted a principal component analysis (Fig. 1).

### Microbial taxa abundance and community composition

Approximately 65 million good quality sequences were obtained after quality control filtering from 24 samples through shotgun metagenomic sequencing (Supplementary Table 2). Of these, 40–45% of the total metagenomic sequences were annotated to a protein of known function using E-value <1 × 10^−5^ and 15-bp minimum alignment length.

Relative abundance of domain and dominant phyla shows large differences between bacteria, archaea, and eukaryotes. Bacterial sequences predominated in every sample (98.36% of all sequences, overall) followed by Archaea (1.21%) and Eukarya (0.43%) (Supplementary Fig. 2a and b). The relative abundance of phyla suggests different patterns of abundance within and between treatments. Functional annotation of major domains reveal different sets of dominant coding genes in each domain (Supplementary Fig. 2c,d). The dominant ones include clustering-based subsystems, carbohydrates and amino acids and their derivatives-related genes.

**Figure 2:**
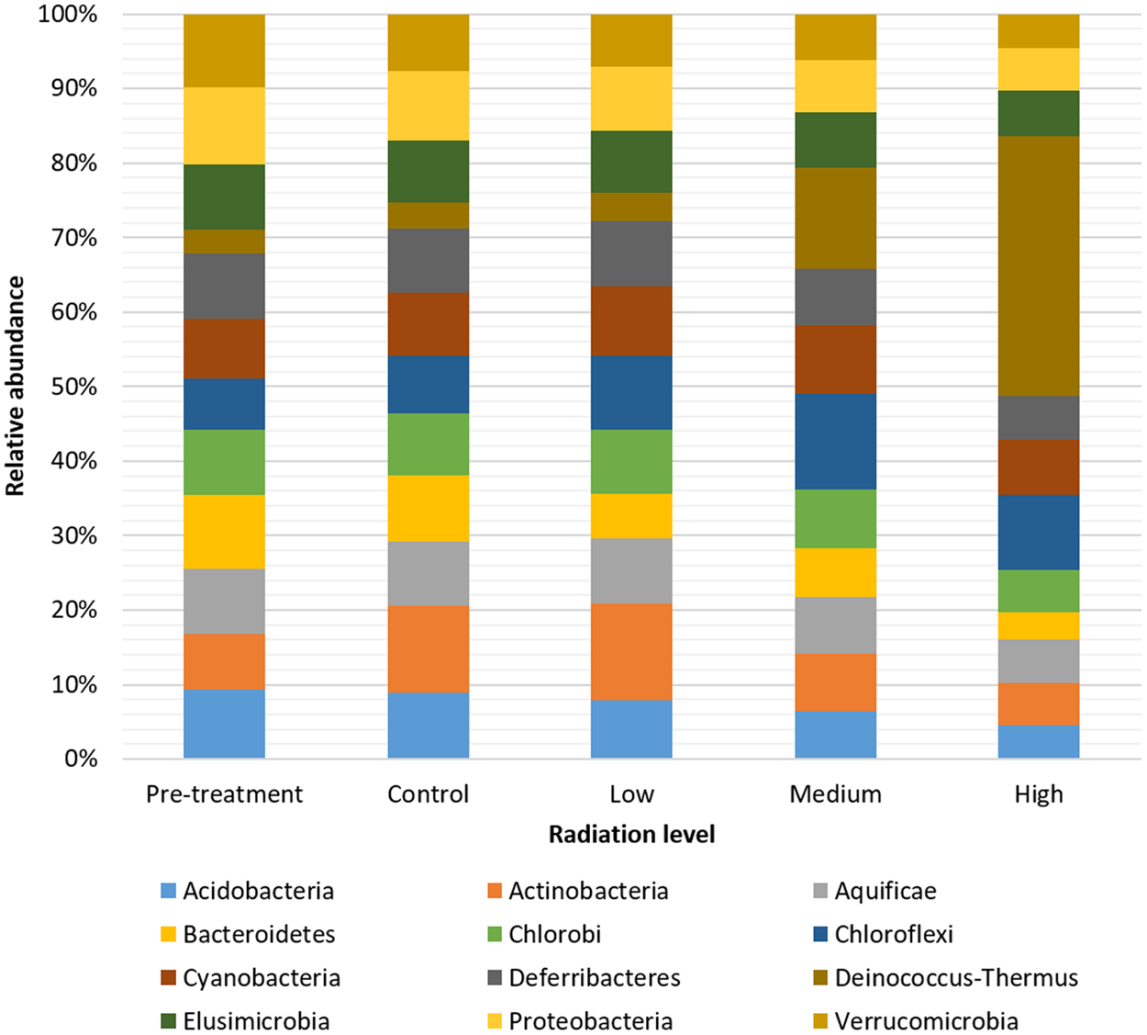
Relative abundance of major recognized bacteria phyla observed in shotgun metagenomics sequence data after exposure to different levels of gamma radiation. The abundance of bacteria phyla in the pre-treatment and control samples differ from the radiation-treated samples. The most abundant phyla in untreated samples was Proteobacteria while Deinococcus-Thermus was the most abundant in the treated samples.

Among the major recognized bacterial phyla recorded in the study, Deinococcus-Thermus was the most abundant, followed by Chloroflexi, Actinobacteria and Proteobacteria (Fig. 2 and Supplementary Fig. 3a). Proteobacteria was the most abundant bacterial phylum in the pre-treatment and control samples whereas Deinococcus-Thermus was the most abundant in the highest irradiation treatment. Functional annotation of these abundant phyla reveal that although the diversity (in terms of number) of coding gene types was similar, they utilized higher proportions of certain types of these genes (Supplementary Fig. 3b).

The most abundant archaeal phylum across all treatments was Nanoarchaeota followed by Thaumarchaeota and Crenarchaeota (Supplementary Fig. 4b). In the control and pre-treatments samples, Euryarchaeota was the most abundant archaeal phylum. This phylum was strongly impacted by the higher doses of irradiation with its far lower abundance in the radiation-treated samples compared to the other phyla (Crenarchaeota, Korarchaeota, Nanoarchaeota and Thaumarchaeota), which all increased their relative abundance. Functional annotation for Nanoarchaeota suggests a high proportion of genes related to DNA processing and protein metabolism (Supplementary Fig. 4e).

Twenty recognizable Eukaryota phyla were obtained (Supplementary Table 3). These included algal, fungal and metazoan phyla (Supplementary Fig. 4a,c,d), which all varied across the treatments. Streptophyta was the most abundant algal phylum in the control samples whereas Chlorophyta and Bacillariophyta were abundant in the radiation treated samples (Supplementary Fig. 4a). Functional annotation of Chlorophyta suggest they were actively utilizing respiration-related genes (Supplementary Fig. 4e). Among the fungal phyla, Ascomycota and Chytridiomycota were most abundant in the control and radiation treated samples respectively (Supplementary Fig. 4c). Chordata was the most abundant metazoa phylum in the control, while Apicomplexa and Echinodermata (presumably mis-assigned and from other metazoan phyla, due to the limitation in metazoan gene characterisation in existing databases) were abundant in the radiation-treated samples (Supplementary Fig. 4d).

The data from qPCR for both bacteria and fungi reveal a decline in gene copy numbers, suggesting a substantial decrease in populations, with increasing radiation dose (Supplementary Fig. 5).

### Functional gene abundance and composition

**Figure 3:**
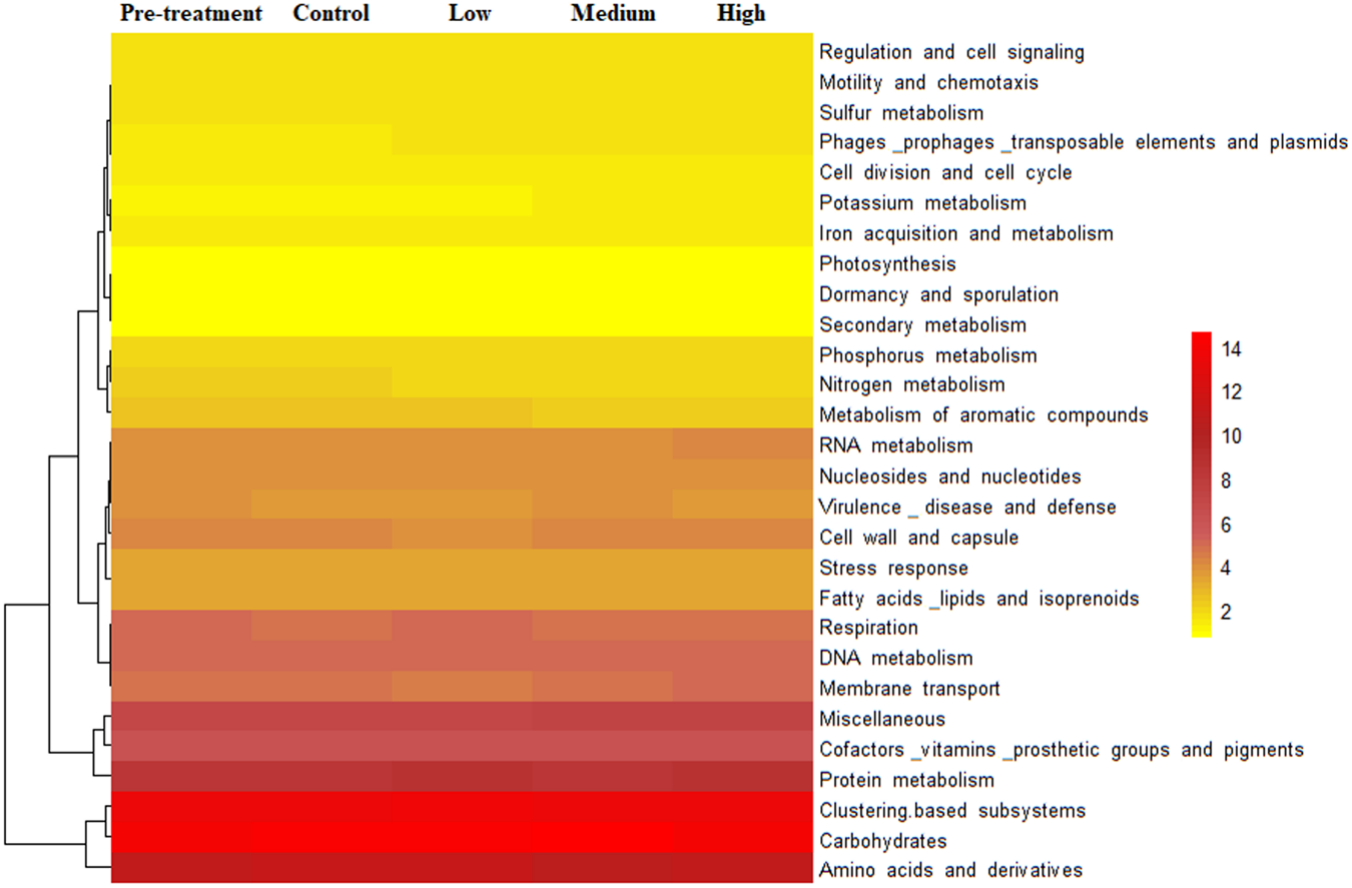
Relative abundance of SEED subsystem level 1 functional genes observed in shotgun metagenomic sequence data after exposure to different levels of gamma radiation. Almost similar pattern of abundance was observed between the control and radiation treated samples.

Twenty-eight functional genes were obtained from SEED level 1 with varying abundance including amino acid derivatives, carbohydrates, cell division and cell cycle, DNA metabolism, dormancy and sporulation, protein metabolism, RNA metabolism, secondary metabolism, virulence and stress response-related genes (Fig. 3 and Table 1). The most abundant gene category across all treatments was carbohydrate-related genes (13.59% on average), followed by clustering based subsystems (12.83%), and genes associated with amino acids and derivatives (10.41%), protein metabolism (7.94%), miscellaneous (6.51%), cofactors, vitamins, prosthetic groups and pigments (5.56%) and DNA metabolism (4.34%) (Fig. 3). Among the 28 functional gene categories, 25 differed significantly across the treatments (Table 1).

**Table 1:** Variations in the relative abundance of SEED subsystem level 1 genes as determined by ANOVA and Kruskal-Wallis test. Of the 28 genes, only three had P_values_ > 0.05 and they include cell division and cell cycle, respiration and secondary metabolism related genes. The non-significant distribution of these genes imply their functions is affected due to exposure to ionizing radiation. On the other hand, significant activities for stress-related genes were obtained along with DNA and RNA metabolisms suggesting ameliorative responses by community to balance the effects of the ionizing radiation.

SEED Level 1 Gene	X^2^ or F	P value	DF
Amino acids and derivatives	21.07	9.06e-07*	4,10
Carbohydrates	18.83	0.00*	4
Cell division and Cell Cycle	0.921	0.47	4,10
Cell wall and capsule	25.68	1.96e-07*	4,10
Clustering based subsystems	15.09	1.04e-05*	4,10
Cofactors, Vitamins, prosthetic group and pigments	15.61	0.00*	4
DNA metabolism	5.155	0.01*	4,10
Dormancy and sporulation	11.25	7.48e-05*	4,10
Fatty acids, lipids and isoprenoids	10.82	9.63e-05*	4,10
Iron acquisition and metabolism	11.82	0.02*	4
Membrane transport	29.91	5.18e-08*	4,10
Metabolism of aromatic compound	17.34	0.00*	4
Miscellaneous	14.12	1.65e-05*	4,10
Motility and chemotaxis	16.38	5.18e-06*	4,10
Nitrogen Metabolism	11.50	0.02*	4
Nucleoside and nucleotides	19.58	1.57e-06*	4,10
Phages, prophages, transposable elements and plasmids	18.03	0.00*	4,10
Phosphorus metabolism	6.345	0.00*	4,10
Photosynthesis	13.31	2.47e-05*	4,10
Potassium metabolism	16.65	0.00*	4
Protein metabolism	7.638	0.00*	4,10
Regulation and cell signaling	3.658	0.02*	4,10
Respiration	1.905	0.15	4,10
RNA metabolism	9.57	0.05*	4
Secondary metabolism	0.503	0.74	4,10
Stress response	6.06	0.00*	4,10
Sulfur metabolism	4.641	0.00*	4,10
Virulence, diseases and defense	18.04	0.00*	4

^*^Significant P_value_ (at P ≤ 0.05).

The gene categories associated with core metabolic functions under adverse conditions such as cell walls and capsules, clustering-based subsystems, DNA metabolism, dormancy and sporulation, virulence and stress response were significantly affected (at P ≤ 0.05) by the treatments (Fig. 3 and Table 1).

### Microbial community and functional diversity

**Figure 4:**
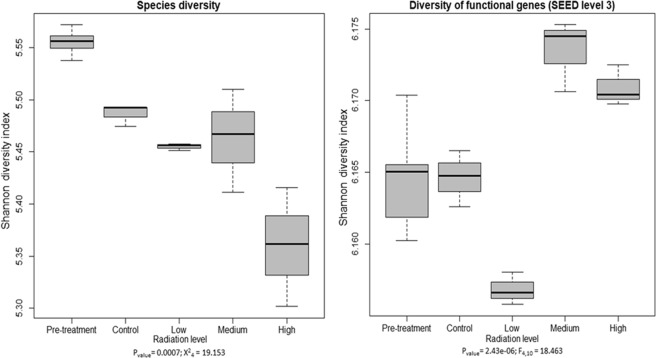
Shannon diversity index of species and functional genes suggest that with increasing radiation species diversity decreases. Functional diversity increased under medium and high irradiation but decreased under low radiation treatment. These were also significantly different at p ≤ 0.05.

All treatments significantly affected the Shannon diversity at all taxonomic and functional gene categorization levels (P ≤ 0.05) (Supplementary Table 4). The species diversity decreased with increasing radiation intensity but interestingly, gene functional diversity (at SEED level three) increased (Fig. 4). The species evenness also decreased with increasing radiation dose, but the species richness increased with increasing radiation (Fig. S6a,b). Functional gene richness also increased with increasing irradiation but the evenness of functional genes showed no significant difference (Supplementary Fig. 6c,d).

**Figure 5:**
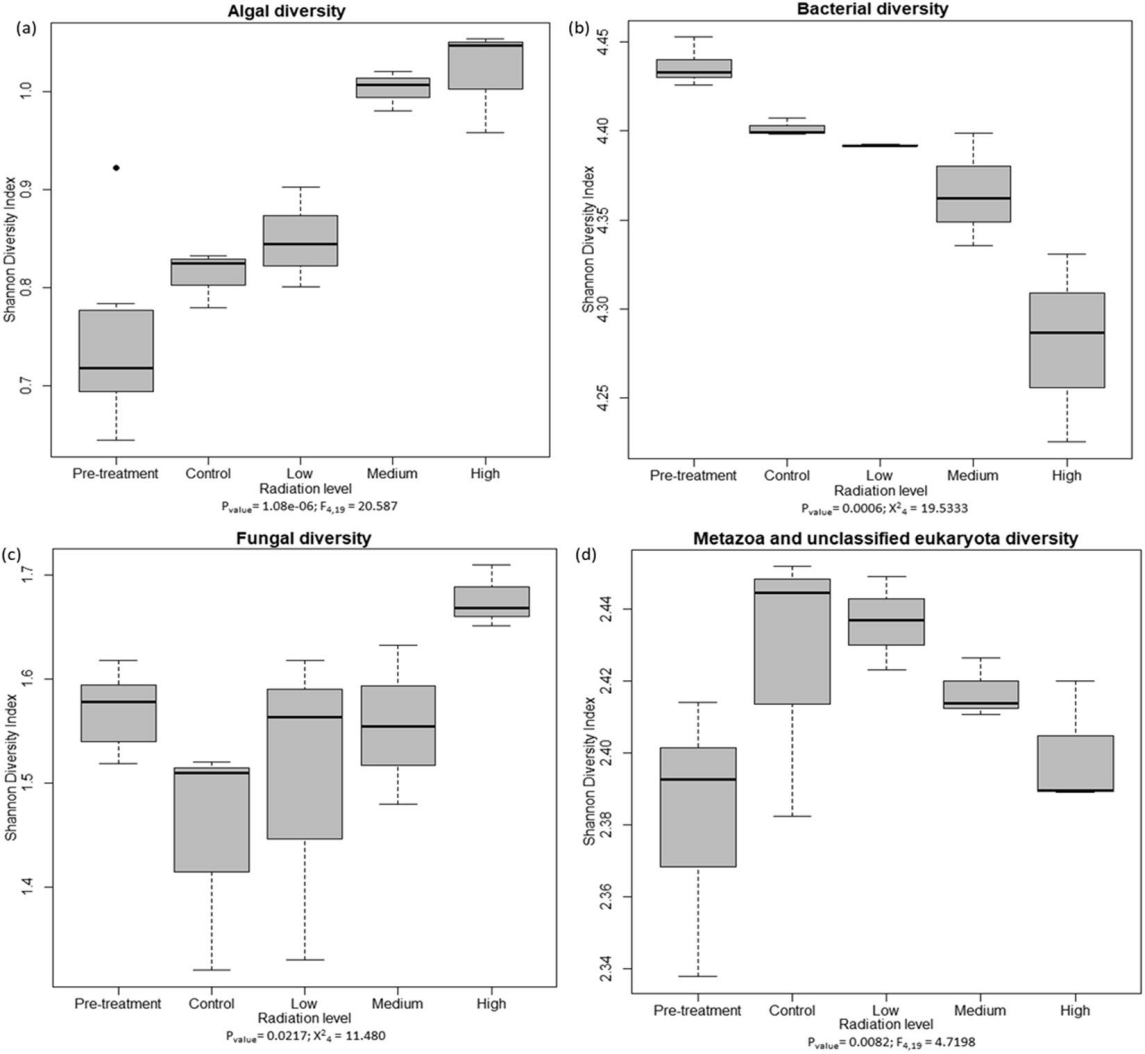
Shannon diversity index for algae (**a**), bacteria (**b**), fungi (**c**) and metazoa (**d**) families reveal alternating patterns in response the radiation treatment. They were all significantly diverse. For algal families, with increasing radiation intensity diversity increased. Fungal diversity was highest under high ionizing radiation intensity. As the intensity of the ionizing radiation increases, bacteria and metazoan diversity decreases.

These effects were further analyzed at the family level, which showed algal diversity increased with increasing radiation intensity, whereas bacterial and metazoan diversity decreased with increasing radiation intensity (Fig. 5a–c). Fungi diversity also increased significantly in response to radiation, although not as strongly as algal diversity (Fig. 5d). In order to understand the effect of treatments on functional diversity within selected categories of genes, we also investigated from SEED level three. The result varied by gene category, for example the diversity either decreased, increased or showed inconsistent patterns with increasing radiation dose (Supplementary Fig. 7a–f).

Rank order nestedness of the samples, reordered in terms of species and functional genes suggests that the radiation treated samples are nested within the pre-treatment and control samples for both taxonomic and functional gene categories at P ≤ 0.0000 (Supplementary Table S5), and thus form a subset of the species and genes of these unirradiated samples.

**Figure 6:**
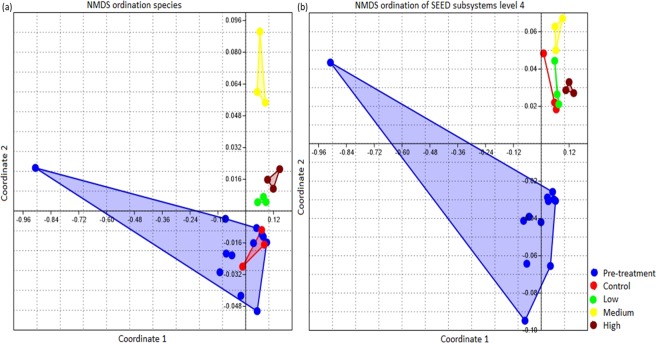
NMDS ordination of species (**a**) and SEED subsystems level 4 (**b**). Radiation treated samples clustered close to each other suggesting more similarity within group than between groups.

To visualize the degree of overall similarity in soil biota taxonomic composition or functional gene composition between the treatment levels, after computation of differences in relative abundance of the different treatment levels, results were ordinated using NMDS. For the taxonomic perspective, the NMDS ordination was generated at the species level (Fig. 6a) and for functional genes at level four of SEED subsystems (Fig. 6b). The samples clustering indicated distinct and repeatable taxonomic and functional genes composition of the soil biota for each treatment. Microbial community and functional diversity co-varied and positively correlated with one another.

## Discussion

Exposure to gamma radiation during the experiment produced a range of differences in both soil biota and soil chemistry, compared to the non-irradiated controls. These changes were clearly dose dependent (Figs 2, 4 and 5).

### Hypothesis 1. Soils exposed to γ-irradiation will have a taxonomically distinct biota with lower diversity compared to untreated control samples

As hypothesized, radiation exposure selected a very distinct soil community. For instance, amongst bacteria (Fig. 2, Supplementary Fig. 3), comparing the control and irradiated soils, there was a shift from dominance by Proteobacteria (in the no-radiation controls) towards Deinococcus-Thermus, which increased with increasing radiation treatments. The ability of Deinococcus-Thermus to resist high doses of ionizing radiation is because of their high DNA repair capacity and resistance to oxidative damage^31,37,58–61^. The absence of this complex DNA repair system in other bacteria groups may have made them less resistant to radiation. However, Chloroflexi, which also lack the same mechanisms as in Deinococcus-Thermus, increased in relative abundance in the irradiated treatments – suggesting that they may be adopting a different strategy to resist γ-irradiation effects. They appear to represent a previously unidentified group of radiation resistors, whose adaptations to survive radiation merit further study.

For archaea, Nanoarchaeota, which increased in abundance (post irradiation) lack DNA polymerases and histone proteins present in Euryarcheota^62^. The absence of these structures been associated with radiation-resistance warrant further investigation. Amongst fungi, radiation exposure brought about a shift from a community dominated by Ascomycota to one dominated by Chytridiomycetes, and Basidiomycota (Fig. 5c). This is in contrasts with the results of simulated space conditions and radiation treatments, where Ascomycota became dominant due to the presence of melanin^63,64^. Soil Ascomycota are generally viewed as rapidly growing forms which exploit nutrient-rich substrates^65^, and their rapid pace of DNA replication may have made them more susceptible to radiation damage. Their reduction here may have presented an opportunity for the even faster growing Chytridiomycota to exploit the dead cellular materials left after each radiation exposure – even if the temporary effects of radiation on their populations may be expected to be more severe. By contrast, the increased relative abundance of Basidiomycota might instead relate to their slower growth rates, on recalcitrant substrates^65,66^. If low rates of DNA replication make their cells less susceptible to radiation damage, then this may explain their preferential survival in the soil.

As anticipated, total species and bacterial diversity detected in the metagenome decreased with increasing radiation intensity (Figs 4 and 5). It is unclear if and when the diversity of a radiation-disturbed disturbed system would return to its pre-disturbance state, and what role local evolution of resistant strains might play in this system^50^. However, in disagreement with our hypothesis, the diversity of fungal and algal (eukaryote) families increased, especially at the highest intensity of γ-irradiation (Fig. 5). Previous results by McNamara *et al*.^50^ suggested that eukaryote (fungal) populations do not recover over periods of weeks after irradiation. The diversity changes we observed may imply either changes in niche competition structure or a change in nutrient availability favouring increased fungal diversity.

### Hypothesis 2. Soils exposed to radiation will have a lower diversity of functional genes

In contradiction with our hypothesis, we observed a greater diversity of functional genes with higher doses of ionizing radiation (Figs 3 and 4). In addition, the diversity of many subcategories of functional genes increased significantly, including those associated with cell wall and capsule, dormancy and sporulation, regulation and cell signalling, stress-related genes, virulence, diseases and defence (Supplementary Fig. 7). This may be an indication that additional functions are required to survive stress from ionizing radiation, or to rapidly exploit opportunities following irradiation while there is little competition and an abundant resource of dead cell material.

The increase in gene function diversity runs opposite to the decreasing total taxonomic diversity of the system. This supports the suggestion of Souza *et al*.^67^ that taxonomic diversity may not necessary be associated with functional diversity. How changes in functional gene diversity influence the overall community functioning is unclear, and further investigation would require empirical measures of soil functions such as catabolic diversity^68,69^.

### Hypothesis 3. Changes in abundance of certain groups of genes associated with radiation exposure

We hypothesized that there would be an increase in the relative abundance of stress response genes, and dormancy and sporulation-related genes, following γ-irradiation. However, only dormancy and sporulation-related genes increased with γ-radiation intensity (Supplementary Fig. 7) even though spore forming groups (such as Proteobacteria and Cyanobacteria) did not increase in abundance under irradiation. Rather, Deinococcus-Thermus and Chloroflexi, which are known to express high levels of dormancy under adverse unfavourable conditions (Fig. 3), increased in abundance. The abundance of dormancy and sporulation-related genes may have increased the survival rates of these bacteria when they were irradiated. These dormancy-related genes are capable of bringing about growth retardation under stress, aiding survival^70^. The decrease in stress-response genes may be related to the taxonomic composition of our system because microbial stress response mechanisms are species-specific and diverse^71^. Therefore, members of the community in our system may be using different tolerance mechanisms in relation to γ-radiation stress, beyond those stress response genes identified in the MG-RAST used in the study. Riley^72^ reported that oxidative stress-related genes are required to act upon the hydroxyl radicals generated by ionizing radiation.

We also predicted a decrease in genes related to competition and cell-cell interactions, following γ-irradiation. Our observations suggest greater importance of interference competition regulating community structure under disturbance from ionizing radiation. It seems then, that under irradiation, cell-cell interactions may be important to survival – in contrast to the prevailing view that stressful environments involve less biotic interaction. One surprising outcome was an increase in the abundance of viruses and transposons in the radiation-treated samples, perhaps due toa breakdown of defense mechanisms in radiation-stressed cells and increased vulnerability to viral attack. Likewise, CRISPR genes – involved in defense against viruses – increased, suggesting greater selection on bacteria from viral attack.

### Changes in soil chemistry

Soil chemical analysis showed that the irradiated soils differed chemically (mainly in soil organic carbon) from the untreated controls, and that the changes were dose dependent (Supplementary Fig. 1). Changes in the soil chemistry of irradiated soils might occur due to perturbations in the biota^73^, or more direct effects of radiation on soil chemistry (with resulting effects on soil biota)^42,74–77^. Dead microorganisms are capable of producing legacy interactions in the soil through their DNA and enzyme actions. It is also known that the use of γ-irradiation in soil sterilization may kill microbes but not inactivate their enzymes, which can leave a legacy in terms of soil matter decomposition through their continued activity^41^. Thus, the changes in soil chemistry between our treatments could be partly due to such effects, analogous to the observation of Jurburg *et al*.^78^ in a microcosm experiment subjected to extreme temperature perturbations.

In our study, the lack of any large changes in pH, even from very high doses, agrees with the results of Bank *et al*.^79^. Together, these results suggest that soil pH may be resistant to change due to γ-irradiation. The decline in soil TC during the time course of the experiment in our non-irradiated samples (Supplementary Fig. 1) is quite rapid and may relate to the initial removal of living roots that had acted as source of short-lived labile carbon^80^. Soil TN was reduced only in the high radiation treatment. This reduction may be due to loss of a large part of the microbial biomass, which represents a large part of the TN in most soils^42,66^. The loss of N may have been a result of leaching of N containing organic or inorganic compounds from the pots, volatilization of ammonia, or dentrification of soil N, following mass death of microbial cells. It is likely, then, that fertility of the soil for plant growth would be affected by this loss of N. Since N is necessary for all microbial cells, it is possible that N limitation would become more significant in the growth and recovery of the soil ecosystem after each irradiation event. There was also an indication in the functional metagenome of decreased activity of both N fixation and ammonia oxidation, with a decline in the relative abundance of genes for both activities and (by inference from the decline in 16 s abundance in qPCR) in absolute abundance.

In conclusion, a range of different changes were found in the soil system following repeated large doses of γ-irradiation. Total bacterial and fungal biomass (indicated by qPCR) in the irradiated soils was much lower than the untreated soil, which implies the capability of the soil system to carry out ecosystem functions is greatly impaired by irradiation. This view is reinforced by the lower TN content of the most highly irradiated soils.

However, the most surprising result of this study was an increase in the finer level (Level 3) functional gene diversity, and in the taxonomic diversity of selected groups of organisms (fungi, and algae). It is unclear why repeated cycles of radiation exposure would produce an increase in taxonomic and functional diversity.

Our study has also pinpointed novel examples of groups of radiation-tolerant groups of organisms: for example the Nanoarchaeota (archaea), and Chytridiomycota (fungi). Possibly some of these can remain active through radiation exposure, whereas others are able to survive in dormant form and then increase rapidly to exploit the aftermath. The radio-tolerance mechanisms of these groups need to be further investigated.

Some caveats need to be underscored as a possible basis for further work. For example, in this study, only one soil was investigated, although this soil is broadly representative in its biota and soil characteristics of moist temperate zone soils in cultivated and disturbed environments^81–83^. As this is apparently the first instance in which a soil has been investigated metagomically in this context, it is unclear how other soils might respond to radiation exposure from the whole biota perspective. However, one might expect that in a less nutrient-rich but organically rich soil, or in a colder environment, the recovery rate of the soil biota between radiation phases would be slower, perhaps with lower taxonomic diversity as fewer forms would be able to maintain their population levels. It is interesting to speculate that in drier or chemically more extreme soil environments, the biota might be adapted (in terms of taxa present or genes) to radiation resistance^84^.

The radiation doses used in our experiment were much higher than most radionuclide-contaminated soils, and it is unclear whether the effects we saw here can be meaningfully back-extrapolated to less heavily irradiated soils. Moreover, the stirring of soil undertaken in this study may have exacerbated the observed effects as suggested by Sheibani & Ahnangar^85^, Wang *et al*.^86^. This includes disturbance of microbial community, soil chemistry and quality.

To rigorously eliminate the possibility that some of our results were derived from ‘legacy’ DNA from cells that died during radiation exposure, one would ideally also employ analysis of cDNA, derived from RNA.

Lastly, we were constrained to running experiments with repeated bursts of radiation exposure. Study under continuous radiation exposure, spanning months or ideally years, might provide findings more relevant to the long-term impacts at contaminated sites or even cumulative effects of radiation in space biology.

## Methods

### Study sites and soil sampling

Due to limitations on the availability of space on the irradiation platform at the radiation facility, we were only able to study one soil. This soil was derived from an early successional environment on the Seoul National University campus (37°27′38.3″N, 126°57′07.5″E), located in the Gwanak Mountain area, South of Seoul, Republic of Korea. The site is characterized by a cool humid temperate climate (MAT 13.3 °C, MAP 1,212.3 mm). The vegetation of the sampling site is composed of around 80% ground cover by common ruderals such as *Poa*, *Festuca*, *Artemisia*, *Taraxacum*, *Senecio* and *Capsella*.

Sampling was carried out in the early winter season (December 2014), when soils were not yet thawed. The soil is a sandy loam typical of that region of Seoul. Three quadrats (each 10 × 10 m in size) were established 50 m apart along a linear transect. Approximately 100 g of soil (from 0 to 10 cm depth) was collected from the four corners and one centre point of the quadrat and pooled to make one individual sample. Overall, 15 samples were collected from within the three quadrats (five samples from each). Thereafter, the collected soil samples were homogenized and sieved with a sieve of 2-mm mesh size and stored at ambient room temperatures for approximately 24 h until they were incubated or irradiated. Using a completely randomized experimental design with five treatments. These were: pre-treatment (initial pre-experiment) soil from before treatments, Control (no radiation exposure but held in the incubator for six weeks) and the Low, Medium, and High radiation after six weeks. There were three replicates for each treatment (except for pre-treatment, which had 12 replicates). For each replicate, 500 g soil was placed in a ceramic self-draining pot.

### Soil incubation and Gamma [^60^Co] radiation treatment

Immediately after sample collection, 100 g of the initial pre-treatment soil sample was used for chemical analysis and DNA extraction. Replicated pots of this pre-treatment soil were also stored untreated for 6 weeks alongside the pots containing the irradiated soils, during the course of the experiment. The soil was stirred once a week in all pots, whether radiation-exposed or not. The stirring was undertaken to ensure that exposure over the whole experiment was fairly evenly distributed and not concentrated on one part of the pot, as gamma ray absorption can result in gradients in radiation exposure within a sample^87^. The free-draining pots were then all stored in an incubator at 25 °C, in randomized positions. We watered each pot with 200 ml deionized water every three days – which was enough to keep the pots close to field capacity in moisture.

The samples for radiation-treatment were each exposed to one of three different levels of ^60^Co gamma radiation treatment at Korea Atomic Energy Research Institute, Daejeon, Korea Republic. The intensities of gamma ^60^Co radiation treatment applied to the soil were low (at 0.1 kGy/hr/wk), medium (at (1 kGy/hr/wk) and high (at 3 kGy/hr/wk). During irradiation, they were held in 500 ml clay pots. The radiation treatment was carried out for one-hour without a break, once a week for six weeks with a cobalt-60 γ-ray irradiator (point source, AECL, IR-79, Canada). Distance between the samples and radiation source was adjusted based on the γ-ray intensity and dose to give a uniform treatment/effect. The soils were then mixed well to ensure homogeneity before the next treatment. Cross contamination was prevented by keeping the pots apart (and not above each other) on the same level, using different sterile rods to stir each pot. Watering was done for each pot separately to prevent splashing. As the soils did not dry out, transmission of dust or dried spores between them was unlikely. The pots were not covered, to allow free gas exchange to the soil microbial community. The positions of replicate pots of different treatments in the incubator were randomized and randomly interchanged each week. The total amount of gamma ^60^Co radiation received by the samples throughout the six-week period was 0.6 kGy (low), 6 kGy (medium) and 18 kGy (high) respectively.

Samples were collected from each pot (of Controls and, Low, Medium and High) after six weeks to be used for soil chemical analysis and total DNA extraction.

### Soil chemical analysis

Samples from each experimental and control replicate on the final day of the study, were analyzed for soil chemistry variables including total organic carbon (TOC), pH, total nitrogen (TN) at National Instrumentation Center for Environmental Management (NICEM, South Korea) based on the standard protocol of the Soil Science Society of America. TOC content was determined by oxidation with 1 N potassium dichromate in acidic medium, according to Rowell and Florence^88^; Rowell^89,90^. pH was determined using a combined pH electrode in a soil-water suspension (soil/tap water = 1:2). TN was determined by sulfuric acid digestion using Se, CuSO4, and K2SO4 as catalysts, with 1 g of soil with final TN content in the digest determined by the regular Kjeldahl distillation method^91^.

### Total DNA extraction and shotgun metagenomic sequencing

The soil DNA was extracted from 0.50 g sample of soil from each sample in replicates, using the Power Soil DNA extraction kit (MoBio Laboratories, Carlsbad, CA, USA) following the protocol described by the manufacturer. DNA isolated from each sample was amplified using primers 338 F (5 = -XXXXXXXXGTACTCCTACGGGAGGCAGCAG-3=) and 533 R (5 = TTACCGCGGCTGCTGGCAC-3 = ), targeting the V3 hypervariable regions^92^. The Polymerase chain reactions (PCR) were carried out under the following thermal profile: denaturation at 94 °C for 2 min, followed by 25 cycles of amplification at 94 °C for 30 s, 57 °C for 30 s and 72 °C for 30 s, followed by a final extension of 72 °C for 5 min. PCR products were analyzed by electrophoresis in 1% agarose gels and were purified using Wizard SV Gel and PCR Clean-up System (Promega, USA). The paired-end sequencing for whole metagenome was carried out using Illumina HiSeq 2000 sequencing system platform (2 × 150 bp) (Illumina) according to the manufacturer’s instructions at Celemics (Celemics, Seoul, Korea). Library preparation, sequencing, and initial quality filtering were performed as described previously^93^.

### Data processing

To annotate the unassembled DNA sequences, we used the Metagenomics Rapid Annotation using Subsystems Technology (MG-RAST) pipeline^94^. The MG-RAST pipeline includes several quality control filtering options for DNA sequence data, including removal of artificial duplicate, reads, and quality-based and length-based read trimming. The M5 non-redundant protein database (M5NR) was used for taxonomic annotation and the SEED database and Clusters of Orthologous Groups database for functional annotation. To identify the sequences, the best BLASTx hit was used with a minimum alignment length of 15 bp and an e-value cut-off of e < 1 × 10−5 and 95% confidence interval. Functional annotation of the most abundant taxa was performed using the filter option. The same was done for select group of genes to reveal the responsible taxa. The shotgun metagenomics sequence data used in this study are deposited in the MG-RAST server under project ID 20322 (http://metagenomics.anl.gov/linkin.cgi?project=mgp20322).

### Quantitative reverse transcription Polymerase Chain Reaction (RT-qPCR)

To investigate the effects of radiation on the absolute abundance of the soil biota, we conducted quantitative real-time PCR (qPCR) amplification of gene copy numbers for two important groups of soil organisms. The qPCR was done on unamplified DNA samples using Applied Biosystems^TM^ QuantStudio^TM^ 6 Flex Real-Time PCR system (by Life Technologies, Carlsbad, CA) to measure the proportion of (1) all bacteria, and (2) all fungi. To quantify bacterial 16S rRNA gene we used the forward primer 5′ TCCTACGGGAGGCAGCAGT-3′ and the reverse primer 5′ -GGACTACCAGGGTATCTAATCCTGTT-3′^95^ and for fungal quantification, we used the primers ITS1F (5′-CTTGGTCATTTAGAGGAAGTAA-3′) and ITS2 (5′-GCTGCGTTCTTCATCGATGC-3′)^96,97^. The reaction mix consisted of 10 μL of 1x QuantiTect SYBR Green master mix (QIAGEN) with HotStar Taq, 0.5 μl of each primer (10 μM), 10 ng DNA template or prepared standard and PCR grade water to a final volume of 20 μL. Thermal cycling conditions were as follows: 50 °C for 2 min, activation step at 95^◦^C for 15 min, followed by 40 cycles of denaturation (95 °C for 15 s), annealing (60 °C for 1 min), and elongation (72 °C for 15 s). All samples including the non-template control and dilution series of standards were run in triplicate. Results were analysed using the ABI Prism 7900HT sequence detection system (Version 2.4). Thereafter, the total gene copy numbers of 16S rRNA and fungal ITS gene copies were calculated for each sample. A 10-fold dilution series were used and PCR amplification efficiency (E) were calculated based on the standard curve using the formula: E = (10^−1/slope^−1) × 100%. No PCR inhibition was found after checking with the method reported by Hospodsky *et al*.^98^.

### Statistical analysis

The taxonomic and functional diversity (Shannon index), richness and evenness were calculated using ‘Vegan’ and ‘BiodiversityR’ packages in R studio^99,100^. To assess the interactions in the samples, we used ANOVA then TukeyHSD posthoc or Kruskal-Wallis then pairwise-Wilcox depending on whether they were normal. Relative abundances were calculated and then used to construct a boxplot or subjected to the ‘pheatmap’ command in R studio. Non-metric dimensional scaling (NMDS) and principal component analysis (PCA) was done using PAST [PAlaeontological Statistic] package^101^ to investigate the relationship of ionizing radiation exposure to the soil chemistry and community composition of each treatment.

Using default input parameters and null model, rank order nestedness was calculated on BINMATNEST^102^ to test whether the community assemblage in each treatment sample is a subset present in another sample. This approach calculates the p-value for rows and column totals and these were reordered following a packed matrix order from high-to-low nestedness as enumerated by Dong *et al*.^103^.

## Supplementary information

Supplementary information

## References

[1] Schwartzbaum JA, Setzer RW, Kupper LL (1994). Exposure to ionizing radiation and risk of cutaneous malignant melanoma: search for error and bias. Ann. Epidemio..

[2] Harrison FL, Anderson SL (1994). Effects of Acute Irradiation on Reproductive Success of the Polychaete Worm, *Neanthes arenaceodentata*. Radiat. Res..

[3] Jones IM (2001). Evaluation of three somatic genetic biomarkers as indicators of low dose radiation effects in clean-up workers of the Chernobyl nuclear reactor accident. Radiat. Prot Dosim..

[4] Robison WL, Concrado CL, Bogen KT, Stoker AC (2003). The effective and environmental half-life of Cs-137 at Coral Island at the former US nuclear test site. J. Environ. Radioact..

[5] Richards ZT, Beger M, Pinca S, Wallace CC (2008). Bikini Atoll coral biodiversity resilience five decades after nuclear testing. Mar. Pollut. Bull..

[6] Martínez A, Matthew C, Romero-Talamás CA, Frías S (2010). An assessment of immediate DNA damage to occupationally exposed workers to low dose ionizing radiation by using the comet assay. Rev. Invest. Clín..

[7] Kerr GD (2013). Workshop report on atomic bomb dosimetry-residual radiation exposure: recent research and suggestions for future studies. Health Phys..

[8] Horneck G, Klaus DM, Mancinelli RL (2010). Space microbiology. Microbiol. Mol. Biol. R..

[9] Horneck, G. *et al*. Viable transfer of microorganisms in the solar system and beyond. (eds Horneck, G. & Baumstark-Khan, C.) *Astrobiology*: *the quest for the conditions of life*. (Springer, Berlin. pp. 57–74. 2002).

[10] Beblo K, Rabbow E, Rachel R, Huber H, Rettberg P (2009). Tolerance of thermophilic and hyperthermophilic microorganisms to desiccation. Extremophiles.

[11] Beblo K, Douki T, Schmalz G (2011). Survival of thermophilic and hyperthermophilic microorganisms after exposure to UV-C, ionizing radiation and desiccation. Arch. Microbiol..

[12] Hollosy F (2002). Effects of ultraviolent radiation on plant cells. Micron.

[13] Kovac E, Keresztes A (2002). Effects of gamma and UV-B/C radiation on plant cells. Micron.

[14] Kujawa J (2004). Effect of low-intensity (3.75-25 J/cm^2^) near-infrared (810nm) laser radiation on red blood cell ATPase activities and membrane structure. J. Clin. Laser Med. Surg..

[15] Cox CS (1989). Airborne bacteria and viruses. Science Progress.

[16] Cabiscol E, Tamarit J, Ros J (2000). Oxidative stress in bacteria and protein damage by reactive oxygen species. Int. Microbiol..

[17] Rothkamm K, Kruger I, Thompson LH, Lobrich M (2003). Pathways of DNA Double-Strand Break Repair during the Mammalian Cell Cycle. Mol. Cell Biol..

[18] Cox MM, Battista JR (2005). *Deinococcus radiodurans* - the consummate survivor. Nat. Rev. Microbiol..

[19] Koonin EV, Wolf YI (2008). Genomics of bacteria and archaea: the emerging dynamic view of the prokaryotic world. Nucleic Acids Res..

[20] Narumi I (2003). Unlocking radiation resistance mechanisms: still a long way to go. TRENDS Microbiol..

[21] Jolivet E, L’Haridon S, Corre E, Forterre P, Prieur D (2003). *Thermococcus gammatolerans* sp. nov., a hyperthermophilic archaeon from a deep-sea hydrothermal vent that resists ionizing radiation. Int. J. Sys. Evol. Microbiol..

[22] Rothschild LJ, Mancinelli RL (2001). Life in extreme environments. Nature.

[23] Daniel R (2005). The metagenomics of soil. Nat. Rev. Microbiol..

[24] Castaneda LE, Barbosa O (2017). Metagenomic analysis exploring taxonomic and functional diversity of soil microbial communities in Chilean vineyards and surrounding native forests. PeerJ.

[25] Sabree, Z. L., Rondon, M. R. & Handelsman, J. Metagenomics. In: *Encyclopedia of Microbiology*. (Academic Press. pp. 622–632. 2009).

[26] Pershina EV (2016). A Comparative Analysis of Microbiomes in Natural and Anthropogenically Disturbed Soils of Northwestern Kazakhstan. Eurasian J. Soil Sci..

[27] Lisitskaya TB, Trosheva TD (2013). Microorganisms stimulating plant growth for sustainable agriculture. Rus. J. Gen. Chem..

[28] Gourmelon V (2016). Environmental and Geographical Factors Structure Soil Microbial Diversity in New Caledonian Ultramafic Substrates: A Metagenomic Approach. PLoS ONE.

[29] Salbu, B. & Skipperud, L. Challenges in Radioecotoxicology. In: *NATO Security through Science Series C: Environmental Security: Multiple* Stressors*:* A *Challenge for the Future*. (eds Mothersill, C., Mosse, I. & Seymour, C.). (Springer Netherland. 3–12p. 2007).

[30] Billi D, Friedmann EI, Hofer KG, Caiola MG, Ocampo-Friedmann R (2000). Ionizing-Radiation Resistance in the Desiccation-Tolerant. Cyanobacterium Chroococcidiopsis. Appl. Environ. Microbiol..

[31] Makarova KS (2001). Genome of the Extremely Radiation-Resistant Bacterium *Deinococcus radiodurans* Viewed from the Perspective of Comparative Genomics. Microbiol. Mol. Biol. Rev..

[32] Errico A, Costanzo V (2010). Differences in DNA replication of unicellular eukaryotes and metazoans: known unknowns. EMBO Rep..

[33] Howland, J. Extremophiles-Microbial Life in Extreme Environments. In: *Biochemical Education* Volume 26 Issue 4. (eds Horikoshi, K. & Grant, W. D.). (Wiley-Liss, Paris, France. 322p. 1998).

[34] de Bruijn, J. F. Stress and environmental regulation of gene expression and adaptation in Bacteria, First Edition. (ed. de Bruijn, F. J.). (John Wiley and Sons, Inc. 2016).

[35] Wexhselbaum RR, Hallahan D, Fuks Z, Kufe D (1994). Radiation induction of immediate early genes: effectors of the radiation-stress response. Int. J. Radiat. Oncol. Biol. Phys..

[36] Little, J. B. Principal Cellular and Tissue Effects of Radiation. In: *Holland-Frei Cancer Medicine*. 6th edition. (eds Decker, B. C. *et al*.). Hamilton (ON). (2003). Available from https://www.ncbi.nlm.nih.gov/books/NBK12344/.

[37] Battista JR (1997). Against all odds: the survival strategies of *Deinococcus radiodurans*. Annu. Rev. Microbiol..

[38] Zhang S, Ye C, Lin H, Lv L, Yu X (2015). UV disinfection induces a Vbnc state in *Escherichia coli* and *Pseudomonas aeruginosa*. Environ. Sci. Technol..

[39] Pianka ER (1970). On r- and K-Selection. Am. Nat..

[40] Andrews, J. H. & Harris, R. F. r- and K-Selection and microbial ecology. In: *Advances in Microbial Ecology*. Volume 9 (ed. Marshall, K. C.). (Springer-Verlag, USA. 99-147pp. 1986).

[41] Blankinship JC, Becerra CA, Schaeffer SM, Schimel JP (2014). Separating cellular metabolism from exoenzyme activity in soil organic matter decomposition. Soil Biol. Biochem..

[42] Eno FC, Popenoe H (1962). The effects of gamma radiation on the availability of Nitrogen and phosphorus in soil. Soil Sci. Am. J..

[43] Eno CF, Popenoe H (1964). Y-Radiation compared with steam and methyl bromide as a soil sterilizing agent. Soil Sci. Soc. Am. Proc..

[44] Mclaren AD (1969). Radiation as a technique in soil biology and biochemistry. Soil Biol. Biochem..

[45] Wolf, D. C. & Skipper, H. D. Soil sterilization. In: *Methods of Soil Analysis*. *Part 2: Microbiological and Biochemical Properties* (eds Weaver, R. W. *et al*.). (Soil Science Society of America, Inc., Madison, WI. pp. 41–51. 1994).

[46] Tuominen L, Kairesalo T, Hartikainen H (1994). Comparison of methods for inhibiting bacterial activity in sediment. Appl. Environ. Microbiol..

[47] Alef, K. & Nannipleri, P. *Methods in Applied Soil Microbiology and Biochemistry*. (Academic Press, San Diego. USA 1995).

[48] Thompson JP (1990). Soil sterilization methods to show VA-mycorrhizae aid P and Zn nutrition of wheat in vertisols. Soil Biol. Biochem..

[49] McNamara NP, Black HIJ, Beresford NA, Parekh NR (2003). Effects of acute gamma irradiation on chemical, physical, and biological properties of soils. Appl. Soil Ecol..

[50] McNamara NP (2007). The sensitivity of a forest soil microbial community to acute gamma-irradiation. Appl. Soil Ecol..

[51] Schimel J, Balser TC, Wallenstein M (2007). Microbial stress-response physiology and its implications for ecosystem function. Ecology.

[52] Liao E-C (2014). Radiation induces senescence and a bystander effect through metabolic alterations. Cell Death Dis..

[53] Adalja, A. A., Toner, E. S., Cicero, A., Fitzgerald, J. & Inglesby, T. V. Radiation at Fukushima: basic issues and concepts, http://www.centerforhealthsecurity.org/resources/cbn/articles/2011/IssueBrief_Radiation-at-Fukushima_03312011.html (2011).

[54] Akashi M (2012). Fukushima Daiichi nuclear accident and radiation exposure. Japan Med Assoc J..

[55] International Atomic Energy Agency. *Present and future environmental impact of the Chernobyl accident*. Waste Safety Section, International Atomic Energy Agency, Vienna, Austria. IAEA-TECDOC-1240. 138p. (2001).

[56] Hassler DM (2013). Mars’ surface radiation environment measured with Mars science laboratory’s Curiosity Rover. Science.

[57] Cockell CS (2018). The UK centre for Astrobiology: a virtual astrobiology centre, accomplishments and lessons learned, 2011–2016. Astrobiology.

[58] Battista JR, Earl AM, Park MJ (1999). Why is *Deinococcus radiodurans* so resistant to ionizing radiation?. TRENDS Microbiol..

[59] Nelson KE, Paulsen IT, Heidelberg JF, Fraser CM (2000). Status of genome projects for nonpathogenic bacteria and archaea. Nat. Biotechnol..

[60] Daly MJ (2004). Accumulation of Mn (II) in *Deinococcus radiodurans* facilitates gamma-radiation resistance. Science.

[61] Daly MJ (2009). A new perspective on radiation resistance based on *Deinococcus radiodurans*. Nat. Rev. Microbiol..

[62] Brochier, C., Gribaldo, S., Zivanovic, Y., Confalonieri, F. & Forterre, P. Nanoarchaea: representatives of a novel archaeal phylum or a fast-evolving euryarchaeal lineage related to Thermococcales? *Genome Biol*. **6**(**5**), Article R42 (2005). 10.1186/gb-2005-6-5-r42 PMC1175954 15892870

[63] Revankar SG, Sutton DA (2010). Melanized Fungi in Human Disease. Clin. Microbiol. Rev..

[64] Blachowicz A (2017). Human presence impacts fungal diversity of inflated lunar/Mars analog habitat. Microbiome.

[65] Pugh GJF (1980). Strategies in fungal ecology. T. Brit. Mycol. Soc..

[66] Colpert JV, Tichelen KKV (1996). Decomposition, nitrogen and phosphorus mineralization from Beech leaf litter colonized by ectomycorrhizal or litter decomposing basidiomycetes. New Phytol..

[67] Souza RC (2016). Shifts in taxonomic and functional microbial diversity with agriculture: How fragile is the Brazilian Cerrado?. BMC Microbiol..

[68] Petchey OL, Gaston KJ (2002). Functional diversity (FD), species richness and community composition. Ecol. Lett..

[69] Torsvik V, Øvreås L (2002). Microbial diversity and function in soil: from genes to ecosystems. Curr. Opin. Microbiol..

[70] Mlynárová L, Nap JP, Bisseling T (2007). The SWI/SNF chromatin-remodeling gene AtCHR12 mediates temporary growth arrest in Arabidopsis thaliana upon perceiving environmental stress. Plant J..

[71] Sévin DC, Stählin JN, Pollak GR, Kuehne A, Sauer U (2016). Global Metabolic Responses to Salt Stress in Fifteen Species. PLoS ONE.

[72] Riley PA (1993). Free radicals in biology: oxidative stress and the effects of ionizing radiation. Int. J. Radiat. Biol..

[73] Wagg. C, Bender SF, Widmer F, van der Heijden MGA (2014). Soil biodiversity and soil community composition determine ecosystem multifunctionality. Proc. Natl. Acad. Sci. USA.

[74] Wainwright M, Killham K, Diprose MF (1980). Effects of 2450MHz microwave radiation on nitrification, respiration and S-oxidation in soil. Soil Biol. Biochem..

[75] Singh A (1997). Increased UV-B radiation reduces N_2_-fixation in tropical leguminous crops. Environ. Pollut..

[76] Rejsek, K., Formanek, P. & Vranova, V. *The Soil Amino Acids: Quality*, *Distribution and Site Ecology* (Nova Science Publishers, New York, NY, USA. 345pp. 2010).

[77] Iheme CI (2016). Effects of Electromagnetic Radiations (EMR) on Some Soil Physicochemical Parameters, Catalase and Dehydrogenase Activities. IOSR J. Environ. Sci. Toxicol. Food Technol..

[78] Jurburg SD (2017). Legacy Effects on the Recovery of Soil Bacterial Communities from Extreme Temperature Perturbation. Front. Microbiol..

[79] Bank TL (2008). Effects of gamma-sterilization on the physico-chemical properties of natural sediments. Chem. Geol..

[80] Fabian J, Zlatanovic S, Mutz M, Premke K (2017). Fungal–bacterial dynamics and their contribution to terrigenous carbon turnover in relation to organic matter quality. ISME J..

[81] Hong, Y. S., Minasny, B., Zhang, Y. S., Kim, Y. H. & Jung, K. H. Digital soil mapping using legacy soil data in Korea. 19th World Congress of Soil Science, Soil Solutions for a Changing World 1–6 August 2010, Brisbane, Australia. pp. 5–8 (2010).

[82] Hwang JY (2000). Mineralogy and chemical composition of the residual soils (Hwangto) from South Korea. J Miner. Soc. Korea..

[83] Igalavithana AD (2017). Assessment of soil health in urban agriculture: soil enzymes and microbial properties. Sustainability.

[84] Goberna MM, Navarro-Cano JA, Valiente-Banuet A, Garcia C, Verdu M (2014). Abiotic stress tolerance and competition-related traits underlie phylogenetic clustering in soil bacterial communities. Ecol. Lett..

[85] Sheibani S, Ahangar AG (2013). Effects of tillage on soil biodiversity. JNAS..

[86] Wang Z, Liu L, Chen Q, Wen X, Liao Y (2016). Conservation tillage increases soil bacteria diversity in the dryland of northern China. Agron. Sustain. Dev..

[87] Smiciklas, I. & Ivanovic, M. S. Chapter 13: *Radioactive contamination of the soil: assessments of pollutants mobility with implication to remediation strategies*. In: Soil Contamination. (eds Larramendy, M. L. & Soloneski, S.). 25pp (2016).

[88] Rowell MJ, Florence ZA (1993). Characteristics associated with differences between undisturbed and industrially-disturbed soil. Soil Biol. Biochem..

[89] Rowell, M. J. Measurement of soil organic matter: A compromise between efficacy and environmental friendliness. *Agricola* **2000**, 66–69 (2000).

[90] Rowell, D. L. *Soil science: methods and applications*. (Routledge, New York, USA 2014).

[91] Thomas, G.W. Exchangeable cations. (eds Page, A. L., Miller, R. H. & Kenny, D. R.). *Method of soil analysis Part-2*. (American Society of Agronomy, Madison, pp. 159–165 1982).

[92] Huse SM (2008). Exploring microbial diversity and taxonomy using SSU rRNA hypervariable tag sequencing. PLoS Genet..

[93] Zhou HW (2011). BIPES, a cost-effective high-throughput method for assessing microbial diversity. ISME J..

[94] Meyer F (2008). The metagenomics RAST server—a public resource for the automatic phylogenetic and functional analysis of metagenomes. BMC Bioinformatics.

[95] Nadkarni MA, Martin FE, Jacques NA, Hunter N (2002). Determination of bacterial load by real-time PCR using a broad-range (universal) probe and primers set. Microbiology.

[96] White, T. J., Bruns, T. D., Lee, S. B. & Taylor, J. W. Amplification and direct sequencing of fungal ribosomal RNA genes for phylogenetics. In: *PCR protocols: a guide to methods and applications*. (eds Innis, M. A., Gelfand, D. H., Sninsky, J. J. & White, T. J.). (Academic Press. pp. 315–322. 1990).

[97] Gardes M, Bruns TD (1993). ITS primers with enhanced specificity for basidiomycetes – application to the identification of mycorrhizae and rusts. Mol. Ecol..

[98] Hospodsky D, Yamamoto N, Peccia J (2010). Accuracy, precision, and method detection limits of quantitative PCR for airborne bacteria and fungi. Appl. Environ. Microbiol..

[99] R Development Core Team. R: a language and environment for statistical computing. R Foundation for Statistical Computing, Vienna, Austria (2008).

[100] Oksanen, J. *et al*. Vegan: community ecology package. R package version 2:3–2. Available at http://cran.rproject.org/web/packages/vegan/index.html (2013).

[101] Hammer, Ø., Harper, D. A. T. & Ryan, P. D. PAST: Paleontological statistics software package for education and data analysis. *Palaeontol*. *Electron*. **4(1)**, 9pp (2001).

[102] Rodríguez-Gironés MA, Santamaría LA (2006). A new algorithm to calculate the nestedness temperature of presence–absence matrices. J. Biogeogr..

[103] Dong K (2016). Soil fungal community development in a high Arctic glacier foreland follows a directional replacement model, with a mid-successional diversity maximum. Sci. Rep..

