# Supplementary material for: Changes in soil taxonomic and functional diversity resulting from gamma irradiation
Source: Sci Rep. 2019 May 27;9:7894. doi: 10.1038/s41598-019-44441-7 (PMC6536540; doi:10.1038/s41598-019-44441-7)
Supplement: Supplementary file 1 — Supplementary information [file 41598_2019_44441_MOESM1_ESM.pdf]

Changes in soil taxonomic and functional diversity resulting from gamma irradiation

Matthew Chidozie Ogwu,[1,2] Dorsaf Kerfahi,[1] HoKyung Song,[1] Ke Dong,[3] Hoseong Seo,[4] Sangyong Lim, [4] Sathiyaraj Srinivasan, [5] Myung Kyum Kim,[5*] Bruce Waldman[1,6] and Jonathan M. Adams[7]*

[1]School of Biological Sciences, Seoul National University, 1 Gwanak-ro, Gwanak-gu, Seoul 08826, Republic of Korea
[2]Department of Plant Biology and Biotechnology, University of Benin, PMB 1154, Ugbowo, Benin City, Edo State, Nigeria
[3]Department of Life Sciences, Kyonggi University, Suwon 443-760, Republic of Korea
[4]Korea Atomic Energy Research Institute, 111 Daedeok-Daero, 989 Beon-Gil, Yuseong-gu, Daejeon, Republic of Korea
[5]Department of Bio and Environmental Technology, Division of Environmental and Life Science, College of Natural Science, Seoul Women's University, 623 Hwarangno, Nowon-gu, Seoul 139-774, Republic of Korea
[6]Department of Integrative Biology, Oklahoma State University, 501 Life Sciences West, Stillwater, Oklahoma, 74078, USA
[7]School of Geographic and Oceanographic Sciences, Nanjing University, Nanjing 210023, Qixia District, Jiangsu Province, People's Republic of China

MCO: matthew.ogwu@uniben.edu, matthew.ogwu@snu.ac.kr
DK: kerfahi.dorsaf@gmail.com
HKS: hk.song15@gmail.com
KD: dongke-007@163.com
HSS: hoseongseo@kaeri.re.kr
SL: saylim@kaeri.re.kr
SS: sathiya.micro@gmail.com
MKS: biotech@swu.ac.kr
BW: waldman@snu.ac.kr
JA: foundinkualalumpur@yahoo.com, geograph.ecol@gmail.com

*** Corresponding author.** Address all correspondence to:
1. Professor Myung Kyum Kim
[Department of Bio and Environmental Technology, Division of Environmental and Life Science, College of Natural Science, Seoul Women's University, 623 Hwarangno, Nowon-gu, Seoul 139-774, Republic of Korea. Email: biotech@swu.ac.kr]
and
2. Professor Jonathan M. Adams.
[School of Geographic and Oceanographic Sciences, Nanjing University, Nanjing 210023, Qixia District, Jiangsu Province, People's Republic of China. Email: foundinkualalumpur@yahoo.com, geograph.ecol@gmail.com. Tel: +8618551988361]

Running title. Soil Metagenome reveals distinct diversity pattern when treated with different levels of ionizing radiation.

Keywords: Dysbiosis, Environmental changes, Gamma radiation, Legacy DNA, Metagenome, Microbial diversity, Microbiome

SUPPLEMENTARY FIGURES AND TABLES

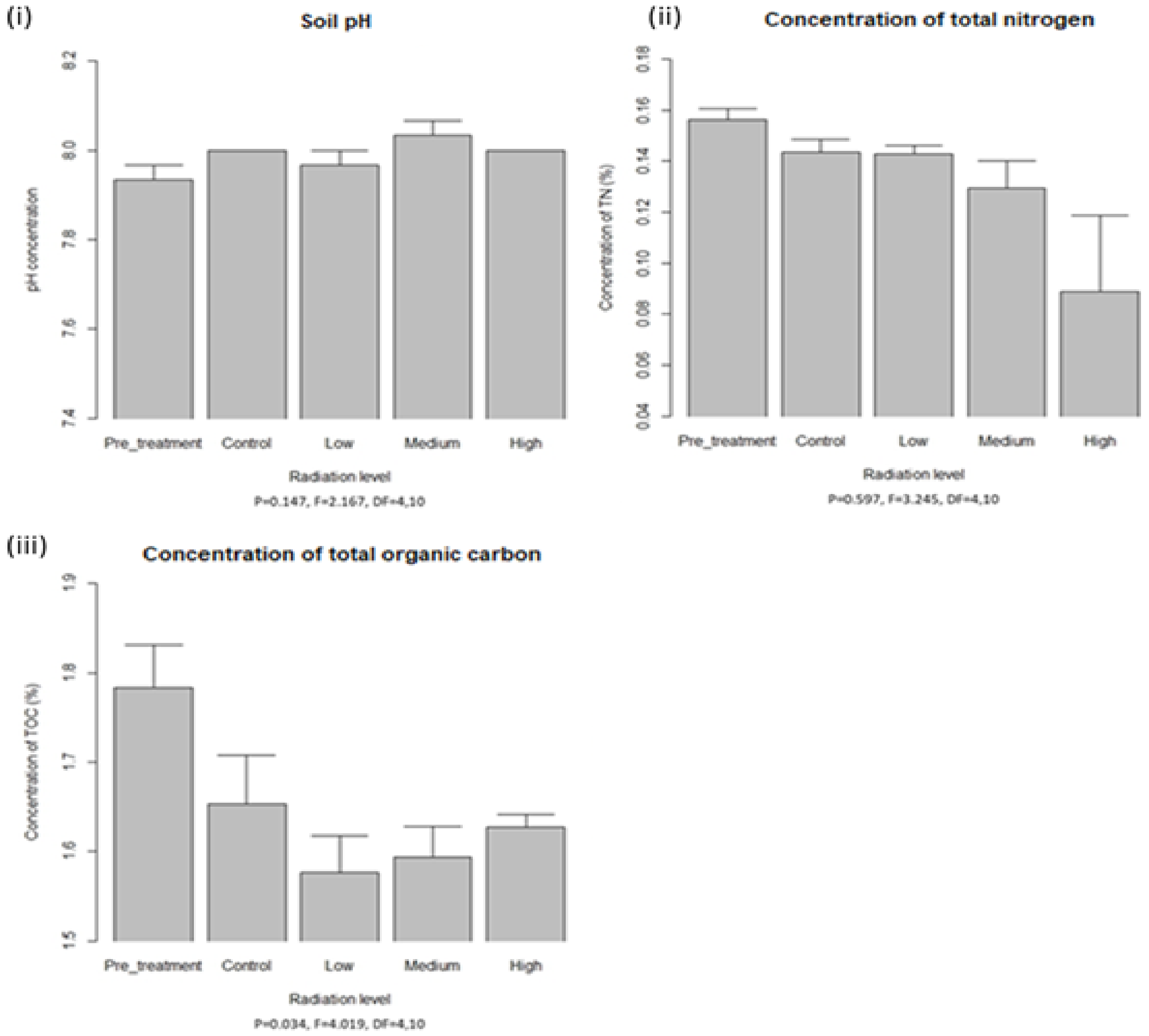

Supplementary Fig. 1. Soil chemical properties.

(a)

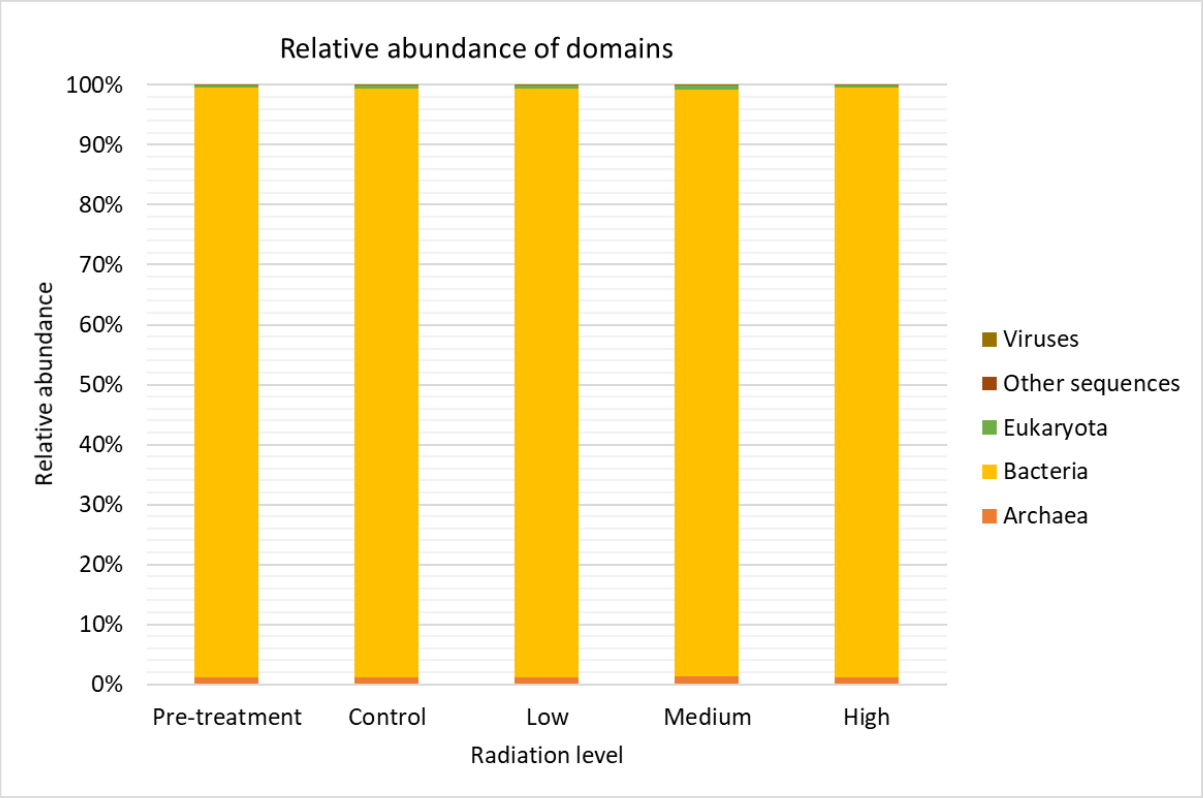

Relative abundance of domains
100%
90%
80%
70%
60%
50%
40%
30%
20%
10%
0%
Relative abundance
Pre-treatment
Control
Low
Medium
High
Radiation level
Viruses
Other sequences
Eukaryota
Bacteria
Archaea

(b).

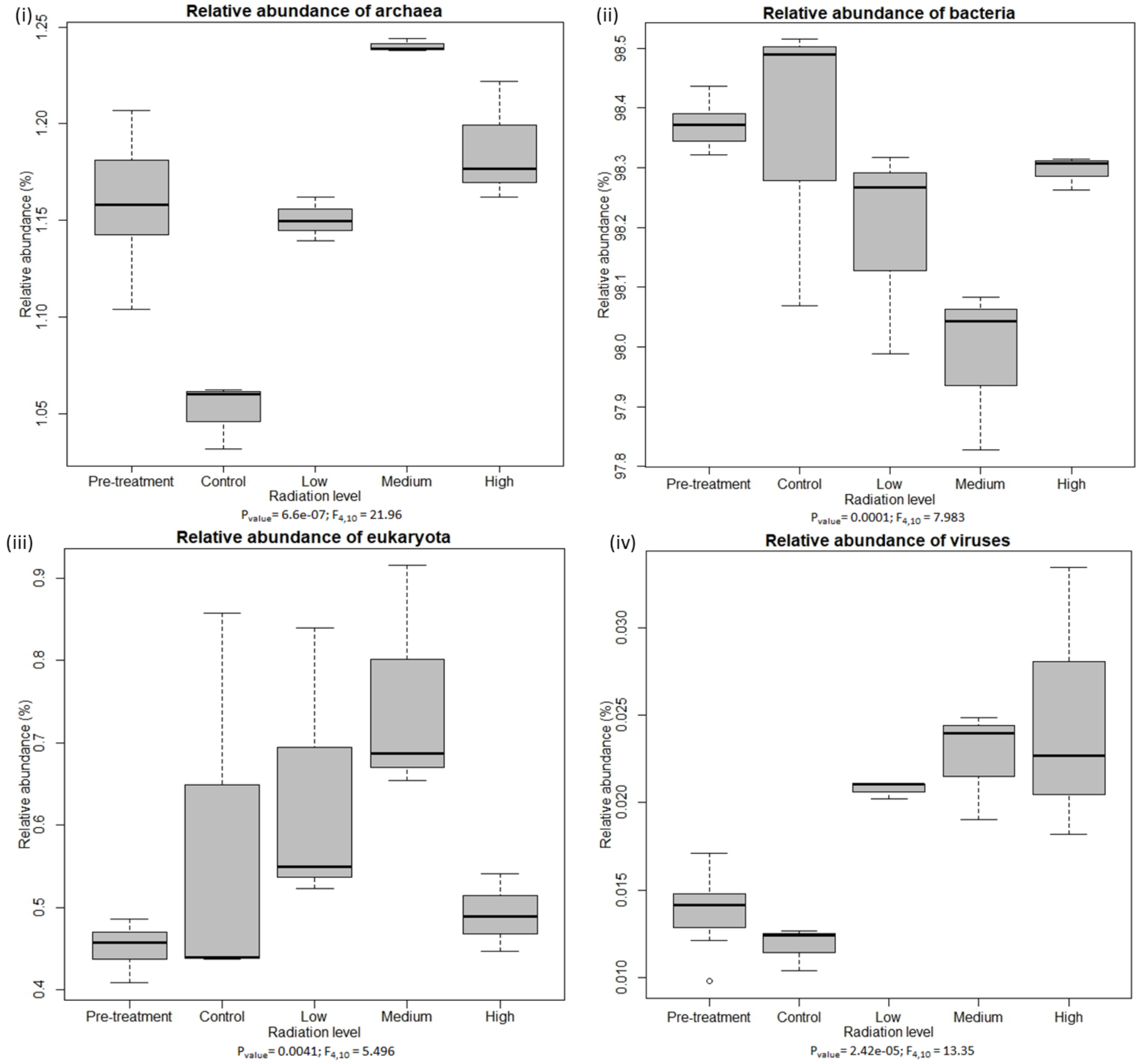

(i)
Relative abundance of archaea
Relative abundance (%)
1.05
1.10
1.15
1.20
1.25
Pre-treatment
Control
Low
Medium
High
Radiation level
P_{value}= 6.6e-07; $F_{4,10}$ = 21.96
(ii)
Relative abundance of bacteria
Relative abundance (%)
97.8
97.9
98.0
98.1
98.2
98.3
98.4
98.5
Pre-treatment
Control
Low
Medium
High
Radiation level
P_{value}= 0.0001; $F_{4,10}$ = 7.983
(iii)
Relative abundance of eukaryota
Relative abundance (%)
0.4
0.5
0.6
0.7
0.8
0.9
Pre-treatment
Control
Low
Medium
High
Radiation level
P_{value}= 0.0041; $F_{4,10}$ = 5.496
(iv)
Relative abundance of viruses
Relative abundance (%)
0.010
0.015
0.020
0.025
0.030
Pre-treatment
Control
Low
Medium
High
Radiation level
P_{value}= 2.42e-05; $F_{4,10}$ = 13.35

(c).

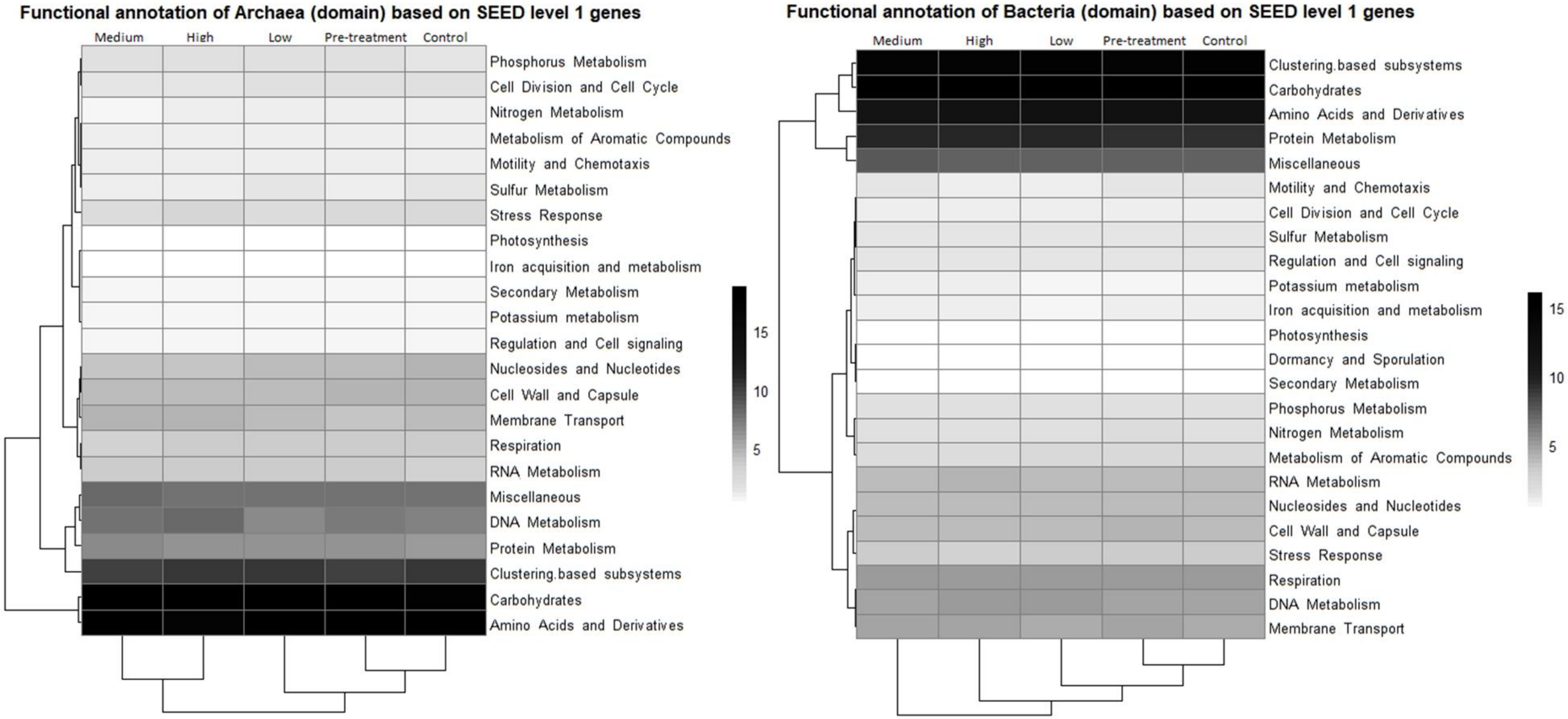

Functional annotation of Archaea (domain) based on SEED level 1 genes
Medium
High
Low
Pre-treatment
Control
Phosphorus Metabolism
Cell Division and Cell Cycle
Nitrogen Metabolism
Metabolism of Aromatic Compounds
Motility and Chemotaxis
Sulfur Metabolism
Stress Response
Photosynthesis
Iron acquisition and metabolism
Secondary Metabolism
Potassium metabolism
Regulation and Cell signaling
Nucleosides and Nucleotides
Cell Wall and Capsule
Membrane Transport
Respiration
RNA Metabolism
Miscellaneous
DNA Metabolism
Protein Metabolism
Clustering.based subsystems
Carbohydrates
Amino Acids and Derivatives
15
10
5
Functional annotation of Bacteria (domain) based on SEED level 1 genes
Medium
High
Low
Pre-treatment
Control
Clustering.based subsystems
Carbohydrates
Amino Acids and Derivatives
Protein Metabolism
Miscellaneous
Motility and Chemotaxis
Cell Division and Cell Cycle
Sulfur Metabolism
Regulation and Cell signaling
Potassium metabolism
Iron acquisition and metabolism
Photosynthesis
Dormancy and Sporulation
Secondary Metabolism
Phosphorus Metabolism
Nitrogen Metabolism
Metabolism of Aromatic Compounds
RNA Metabolism
Nucleosides and Nucleotides
Cell Wall and Capsule
Stress Response
Respiration
DNA Metabolism
Membrane Transport
15
10
5

(d).

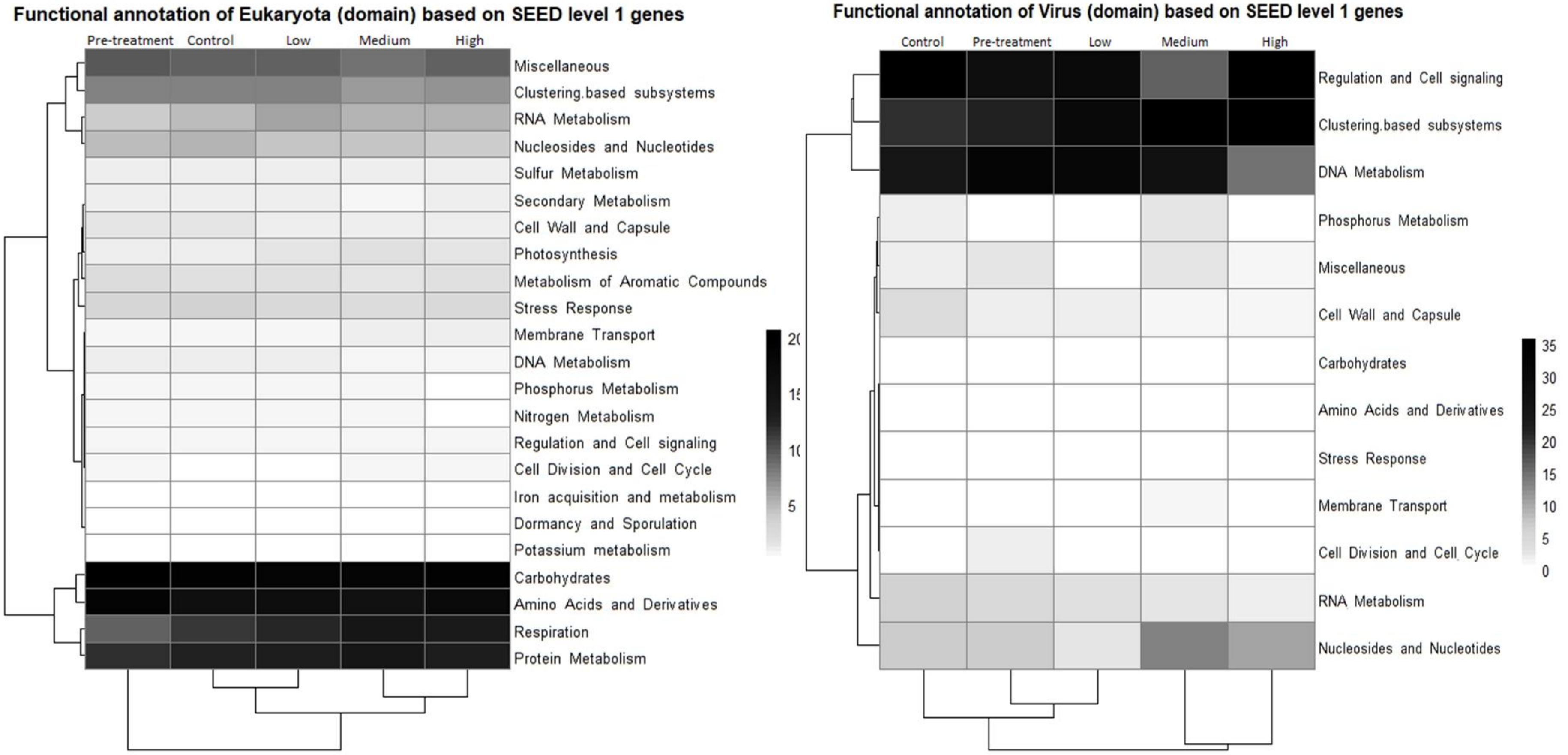
Functional annotation of Eukaryota (domain) based on SEED level 1 genes
Pre-treatment
Control
Low
Medium
High
Miscellaneous
Clustering.based subsystems
RNA Metabolism
Nucleosides and Nucleotides
Sulfur Metabolism
Secondary Metabolism
Cell Wall and Capsule
Photosynthesis
Metabolism of Aromatic Compounds
Stress Response
Membrane Transport
DNA Metabolism
Phosphorus Metabolism
Nitrogen Metabolism
Regulation and Cell signaling
Cell Division and Cell Cycle
Iron acquisition and metabolism
Dormancy and Sporulation
Potassium metabolism
Carbohydrates
Amino Acids and Derivatives
Respiration
Protein Metabolism
5
Functional annotation of Virus (domain) based on SEED level 1 genes
Control
Pre-treatment
Low
Medium
High
Regulation and Cell signaling
Clustering.based subsystems
DNA Metabolism
Phosphorus Metabolism
Miscellaneous
Cell Wall and Capsule
Carbohydrates
Amino Acids and Derivatives
Stress Response
Membrane Transport
Cell Division and Cell Cycle
RNA Metabolism
Nucleosides and Nucleotides
35
30
25
20
15
10
5
0

Supplementary Fig. 2. Relative abundance of domains observed in shotgun metagenomic sequence data after exposure to different levels of gamma radiation. The most abundant domain is Bacteria followed by Archaea (a). The domains were all significantly different at $p \leq 0.05$ (b). The relative abundance of Archeae increased while that of Bacteria decreased with increasing irradation, whereas the relative abundance of Eukaryota and Viruses increased in low and medium treatment but reduced under high treatment doses (b). The functional annotation of these major domains reveal different set of dominant genes may be responsible for their abundance (c and d). To varying degrees, Bacteria and Archaea were sustained by clustering based subsystems, carbohydrates as well as amino acids and derivatives related genes depending on the radiation intensity (c). Carbohydrate and Amino acids were the major genes utilized by Eukaryotes while Viruses were sustained by clustering based susbsystems, regulation and cell signalling as well as by DNA metabolism related genes (d).

(a).

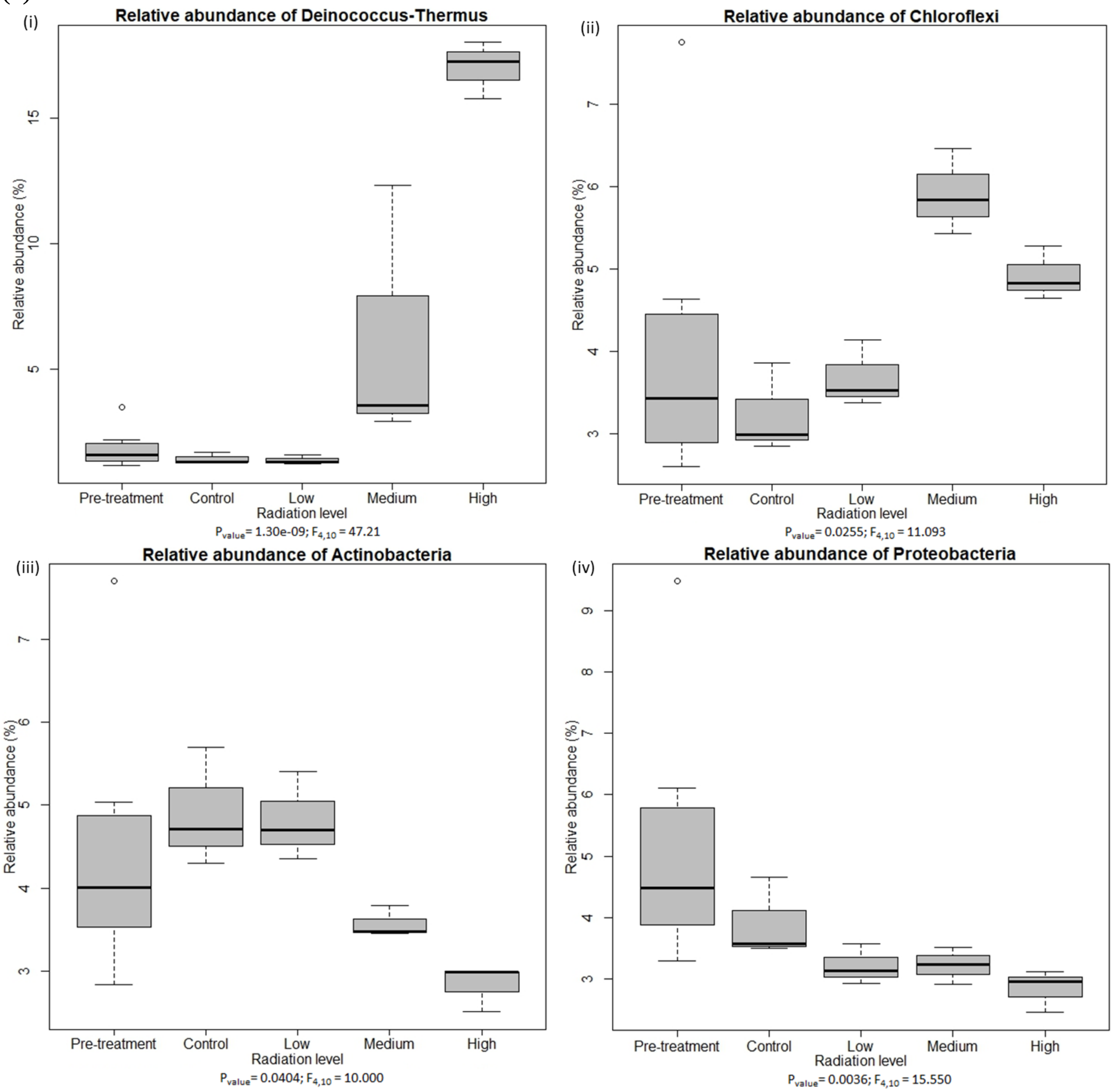
(i)
Relative abundance of Deinococcus-Thermus
Relative abundance (%)
5
10
15
Pre-treatment
Control
Low
Medium
High
Radiation level
P_value = 1.30e-09; F_4,10 = 47.21
(ii)
Relative abundance of Chloroflexi
Relative abundance (%)
3
4
5
6
7
Pre-treatment
Control
Low
Medium
High
Radiation level
P_value = 0.0255; F_4,10 = 11.093
(iii)
Relative abundance of Actinobacteria
Relative abundance (%)
3
4
5
6
7
Pre-treatment
Control
Low
Medium
High
Radiation level
P_value = 0.0404; F_4,10 = 10.000
(iv)
Relative abundance of Proteobacteria
Relative abundance (%)
3
4
5
6
7
8
9
Pre-treatment
Control
Low
Medium
High
Radiation level
P_value = 0.0036; F_4,10 = 15.550

(b).

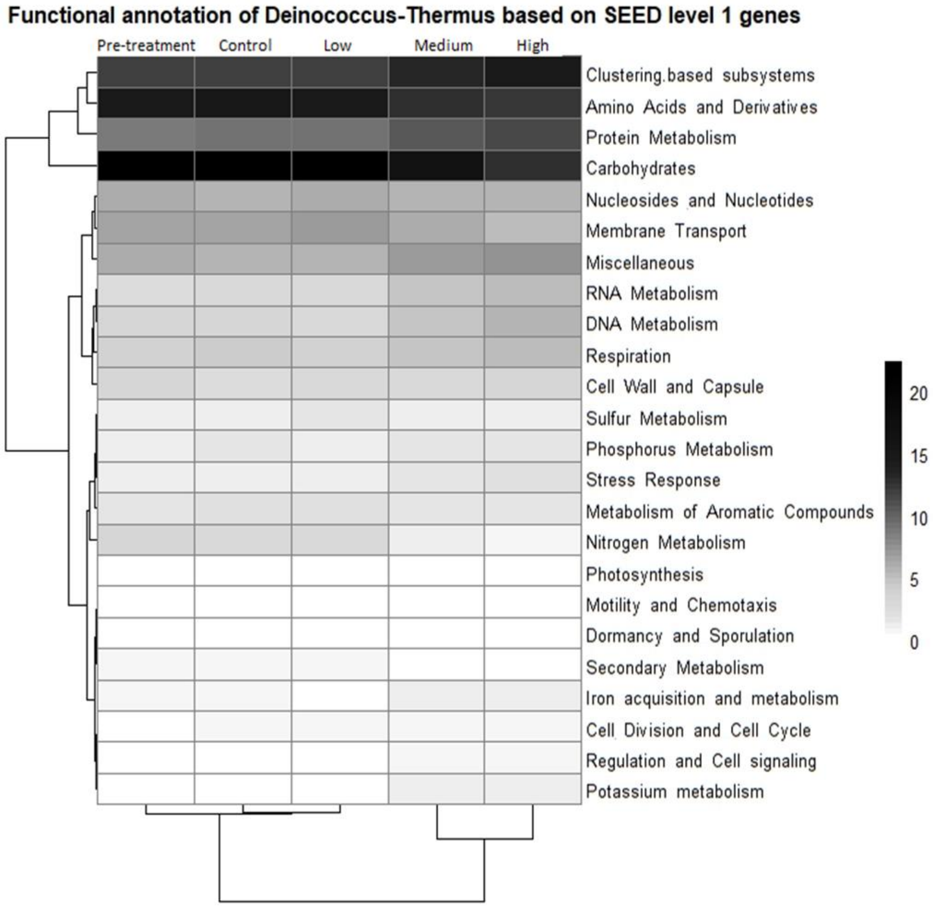
Functional annotation of Deinococcus-Thermus based on SEED level 1 genes
Pre-treatment
Control
Low
Medium
High
Clustering.based subsystems
Amino Acids and Derivatives
Protein Metabolism
Carbohydrates
Nucleosides and Nucleotides
Membrane Transport
Miscellaneous
RNA Metabolism
DNA Metabolism
Respiration
Cell Wall and Capsule
Sulfur Metabolism
Phosphorus Metabolism
Stress Response
Metabolism of Aromatic Compounds
Nitrogen Metabolism
Photosynthesis
Motility and Chemotaxis
Dormancy and Sporulation
Secondary Metabolism
Iron acquisition and metabolism
Cell Division and Cell Cycle
Regulation and Cell signaling
Potassium metabolism
20
15
10
5
0

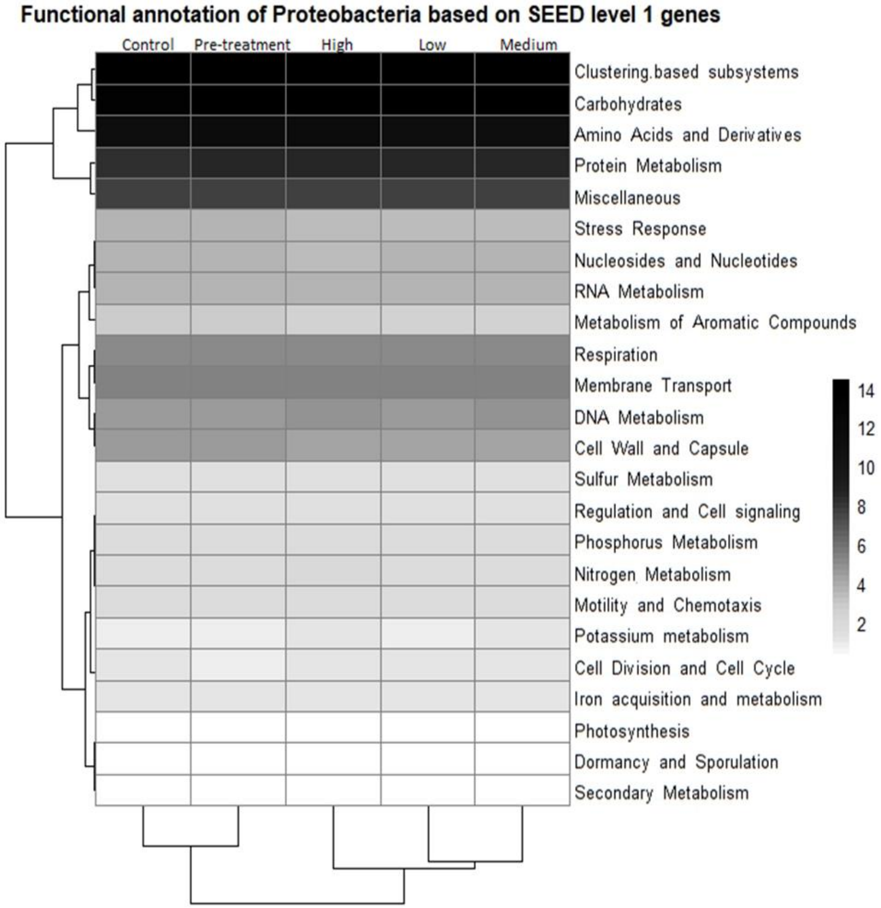
Functional annotation of Proteobacteria based on SEED level 1 genes
Control
Pre-treatment
High
Low
Medium
Clustering.based subsystems
Carbohydrates
Amino Acids and Derivatives
Protein Metabolism
Miscellaneous
Stress Response
Nucleosides and Nucleotides
RNA Metabolism
Metabolism of Aromatic Compounds
Respiration
Membrane Transport
DNA Metabolism
Cell Wall and Capsule
Sulfur Metabolism
Regulation and Cell signaling
Phosphorus Metabolism
Nitrogen Metabolism
Motility and Chemotaxis
Potassium metabolism
Cell Division and Cell Cycle
Iron acquisition and metabolism
Photosynthesis
Dormancy and Sporulation
Secondary Metabolism
14
12
10
8
6
4
2

Supplementary Fig. 3. The relative abundance of most abundant bacteria phyla across all treatment levels (a). The functional annotation of the most abundant phyla in the control and radiation treatment reveal that although the diversity (in terms of numbers) of genes were similar the major group differ in abundance. Deinococcus-Thermus had greater annotation for carbohydrates while it was clustering based subsystems related genes in Proteobacteria (b).

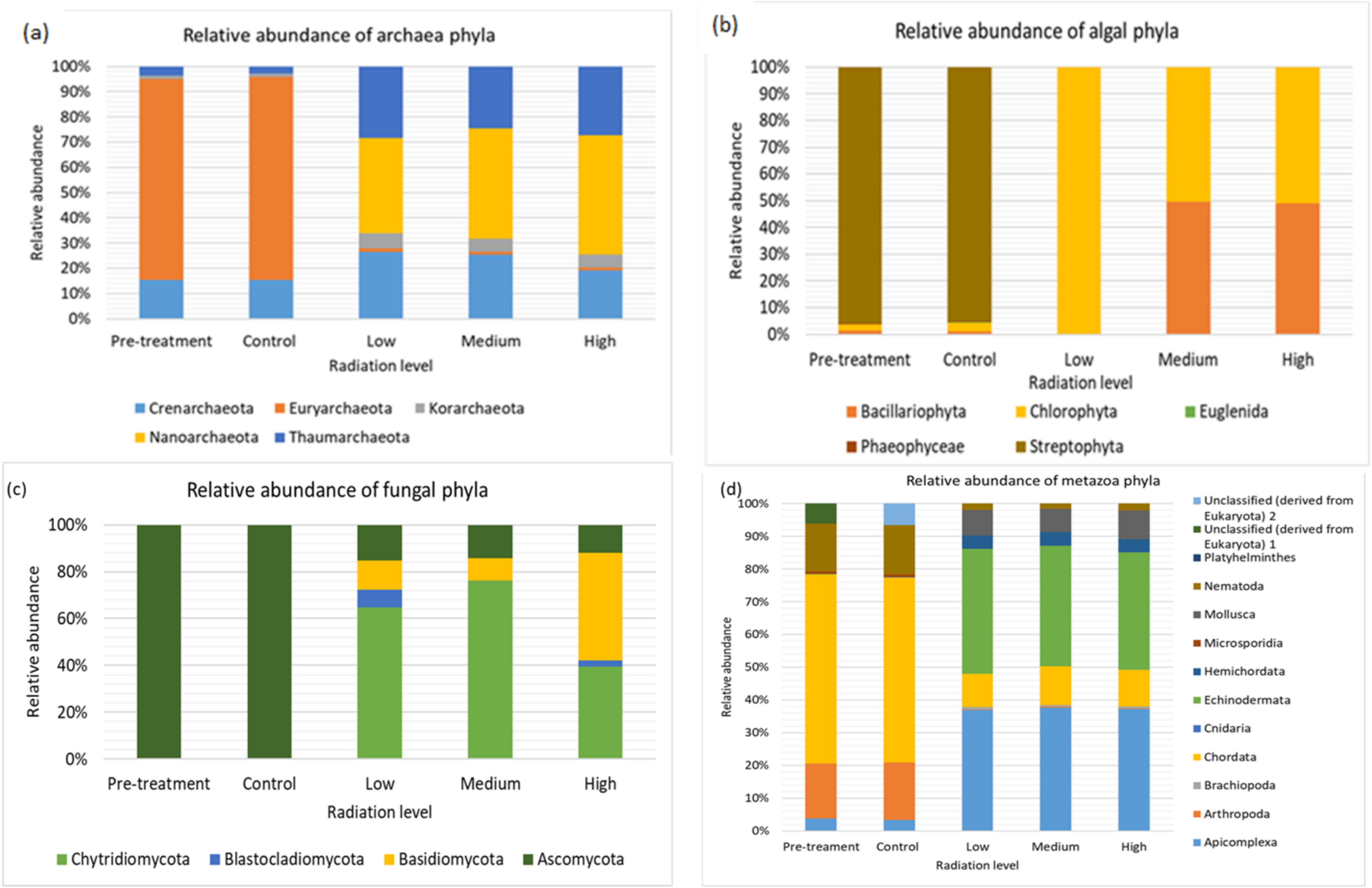
(a)
Relative abundance of archaea phyla
Relative abundance
100%
90%
80%
70%
60%
50%
40%
30%
20%
10%
0%
Pre-treatment
Control
Low
Medium
High
Radiation level
Crenarchaeota
Euryarchaeota
Korarchaeota
Nanoarchaeota
Thaumarchaeota
(b)
Relative abundance of algal phyla
Relative abundance
Pre-treatment
Control
Low
Medium
High
Radiation level
Bacillariophyta
Chlorophyta
Euglenida
Phaeophyceae
Streptophyta
(c)
Relative abundance of fungal phyla
Relative abundance
100%
80%
60%
40%
20%
0%
Pre-treatment
Control
Low
Medium
High
Radiation level
Chytridiomycota
Blastocladiomycota
Basidiomycota
Ascomycota
(d)
Relative abundance of metazoa phyla
Relative abundance
Pre-treament
Control
Low
Medium
High
Radiation level
Unclassified (derived from Eukaryota) 2
Unclassified (derived from Eukaryota) 1
Platyhelminthes
Nematoda
Mollusca
Microsporidia
Hemichordata
Echinodermata
Cnidaria
Chordata
Brachiopoda
Arthropoda
Apicomplexa

(e).

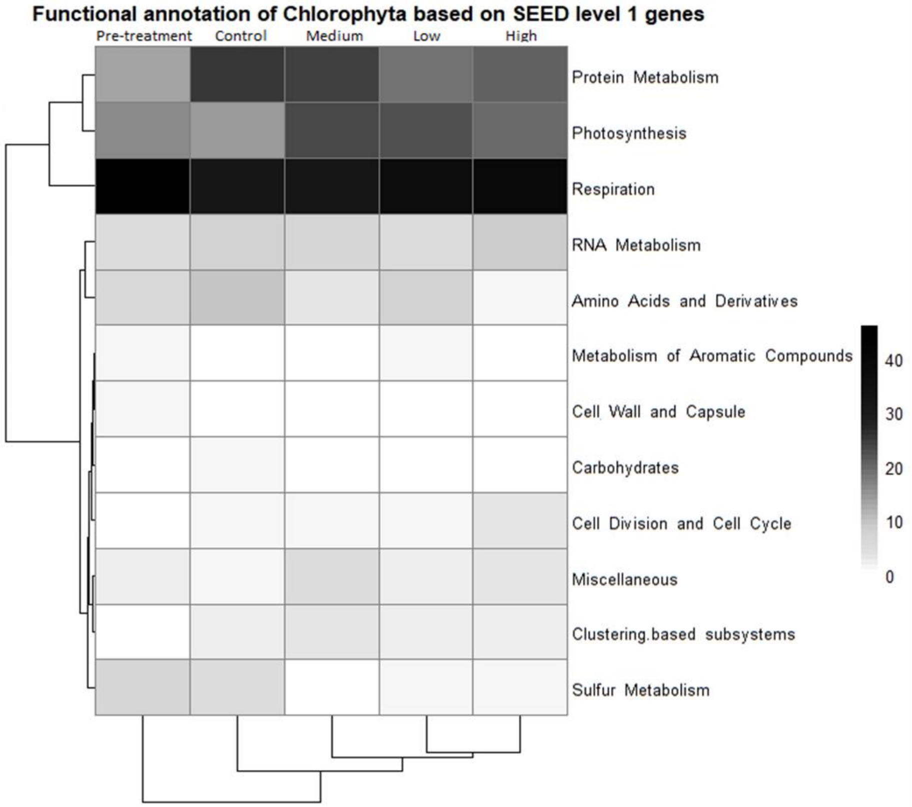
Functional annotation of Chlorophyta based on SEED level 1 genes
Pre-treatment
Control
Medium
Low
High
Protein Metabolism
Photosynthesis
Respiration
RNA Metabolism
Amino Acids and Derivatives
Metabolism of Aromatic Compounds
Cell Wall and Capsule
Carbohydrates
Cell Division and Cell Cycle
Miscellaneous
Clustering.based subsystems
Sulfur Metabolism
40
30
20
10
0

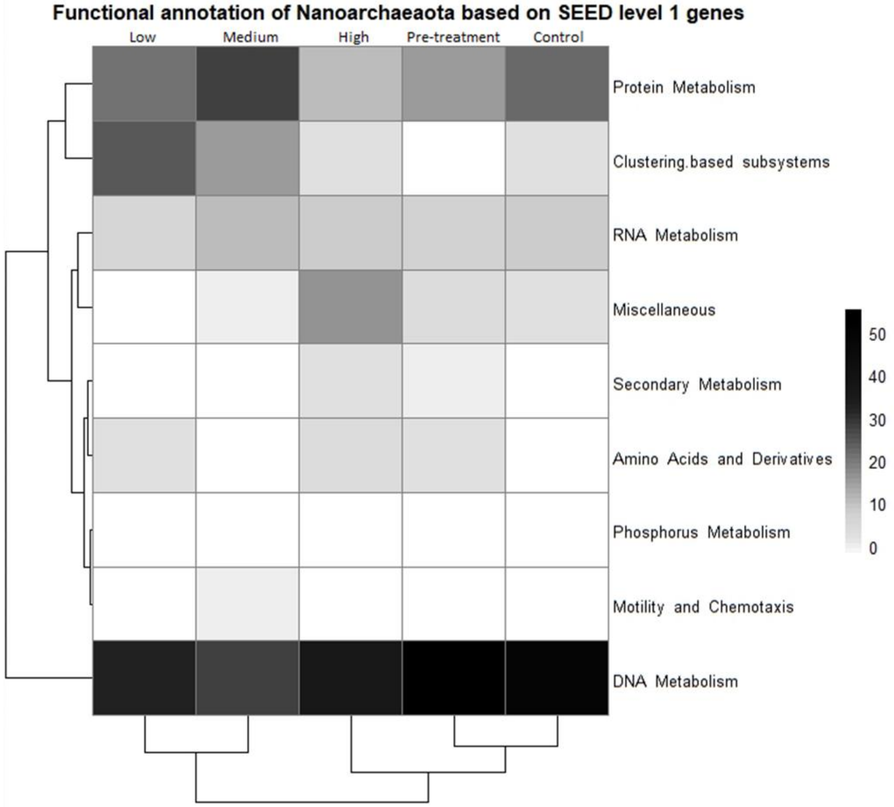
Functional annotation of Nanoarchaeaota based on SEED level 1 genes
Low
Medium
High
Pre-treatment
Control
Protein Metabolism
Clustering.based subsystems
RNA Metabolism
Miscellaneous
Secondary Metabolism
Amino Acids and Derivatives
Phosphorus Metabolism
Motility and Chemotaxis
DNA Metabolism
50
40
30
20
10
0

(f).

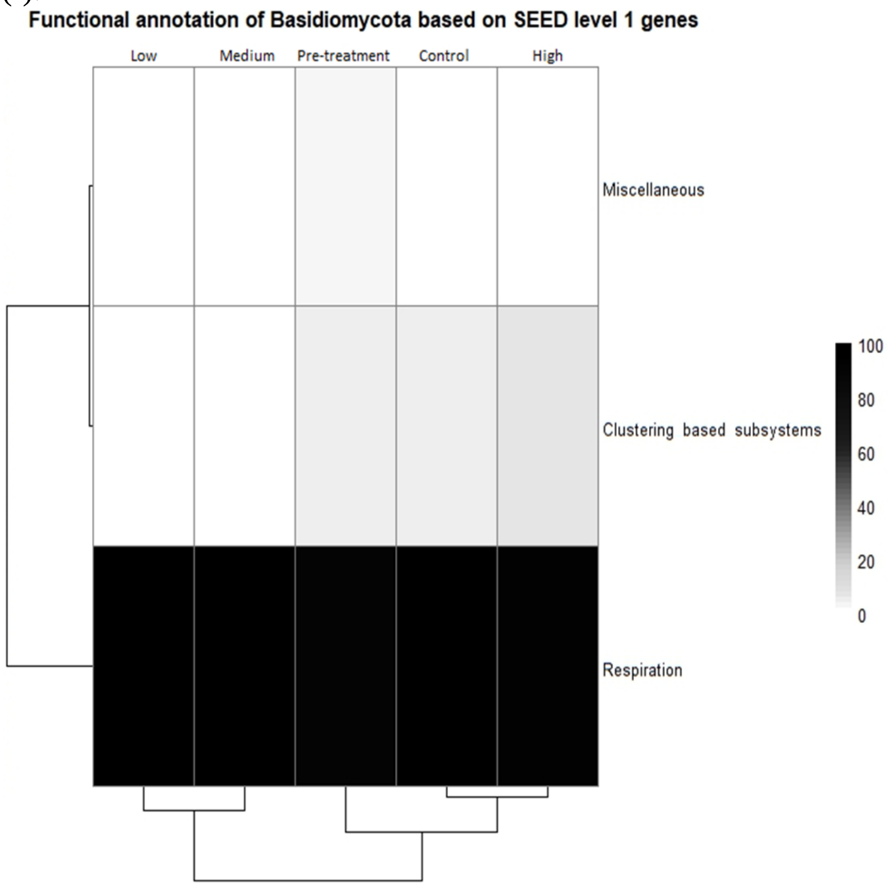

Functional annotation of Basidiomycota based on SEED level 1 genes
Low
Medium
Pre-treatment
Control
High
Miscellaneous
Clustering based subsystems
Respiration
100
80
60
40
20
0

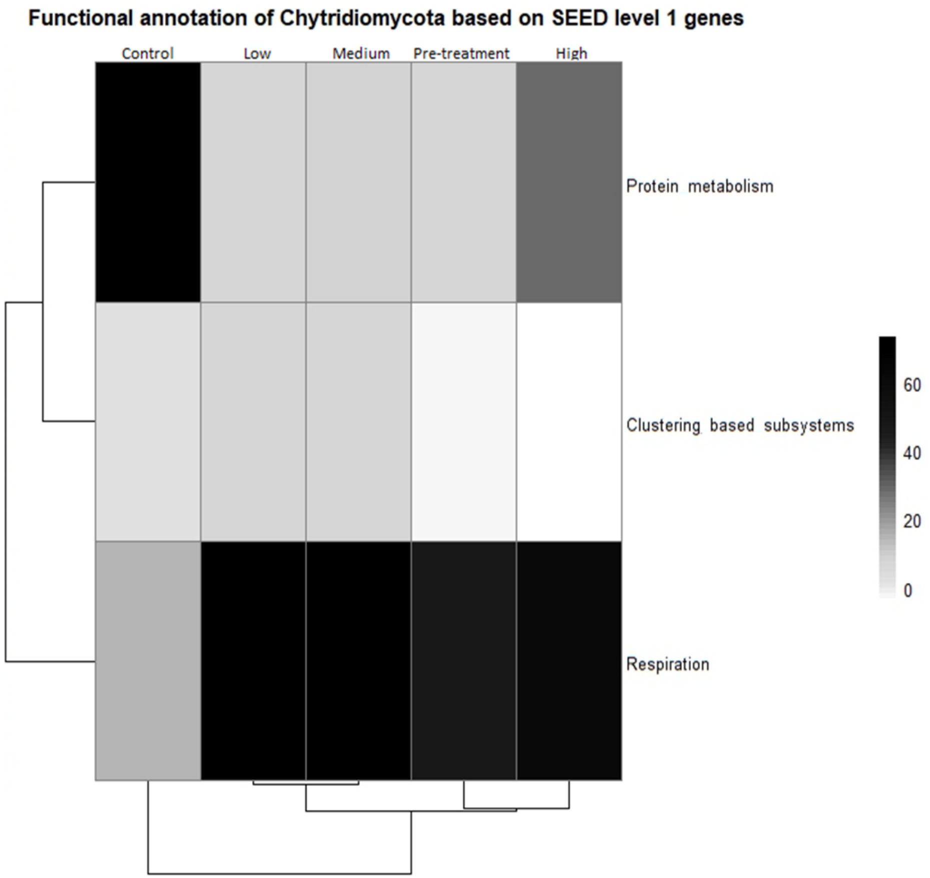

Functional annotation of Chytridiomycota based on SEED level 1 genes
Control
Low
Medium
Pre-treatment
High
Protein metabolism
Clustering based subsystems
Respiration
60
40
20
0

Supplementary Fig. 4. Relative abundance of Archaea and algal, fungal and metazoa phyla observed in shotgun metagenomic sequence data after exposure to different levels of gamma radiation. A pronounced difference is observed in the abundance of all the phyla between the treated and control samples (Fig. 5a-d). The most abundant archaea phylum in the pre-treatment and control samples is Euryarchaeota and Nanoarchaeota in the samples exposed to ionizing radiation (b). Streptophyta was the most abundant algal phylum in the control samples whereas Chlorophyta and Bacillariophyta were abundant in the radiation treated samples (a). Among the fungal phyla, Ascomycota and Chytridiomycota were most abundant in the control and radiation treated samples respectively (c). Chordata was the most abundant metazoa phylum in the control while Apicomplexa and Echinodermata were abundant in the radiation treated samples (d). Functional annotation of the most abundant group of Eukaryota suggest they utilized mostly respiration related genes while Nanoarchaeota had high amount of DNA and protein metabolism related genes (e and f).

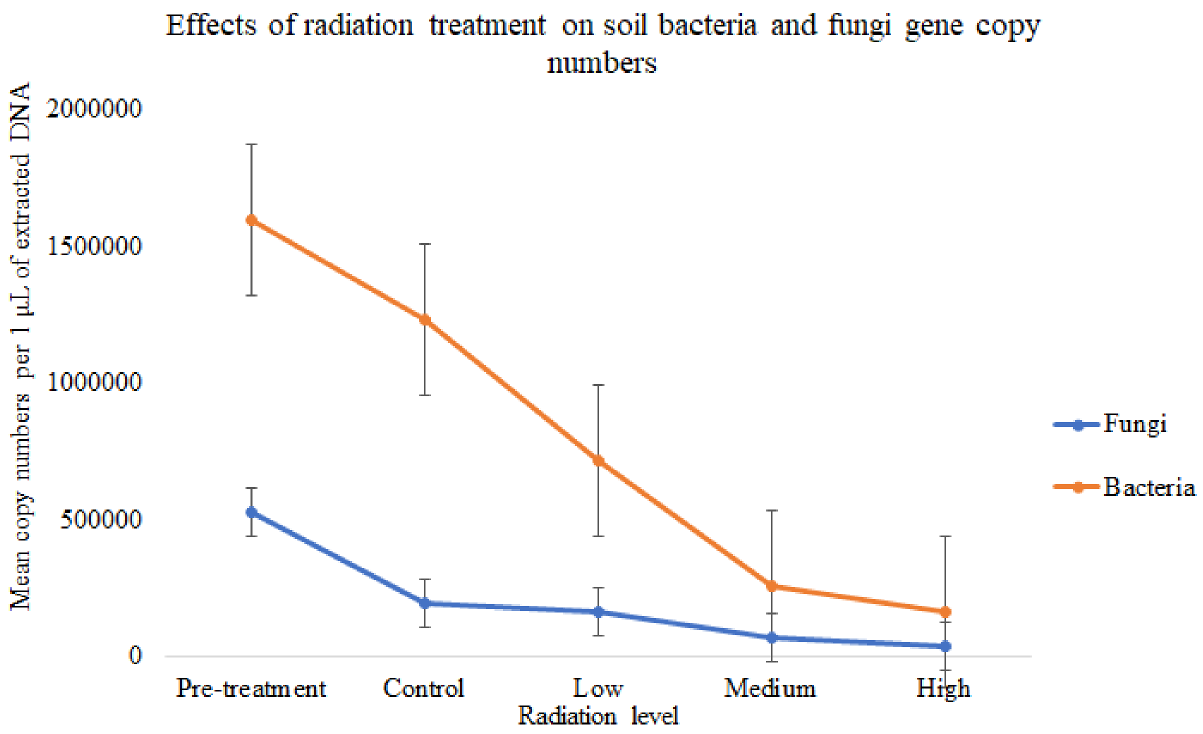

Supplementary Fig. 5. Effects of radiation treatment on soil bacteria and fungi gene copy numbers. The bacteria gene copy number was higher than those of the fungal but both showed similar patterns. Pre-treatment sample had the highest copy numbers whereas the radiation treated samples decreased with increasing radiation dose.

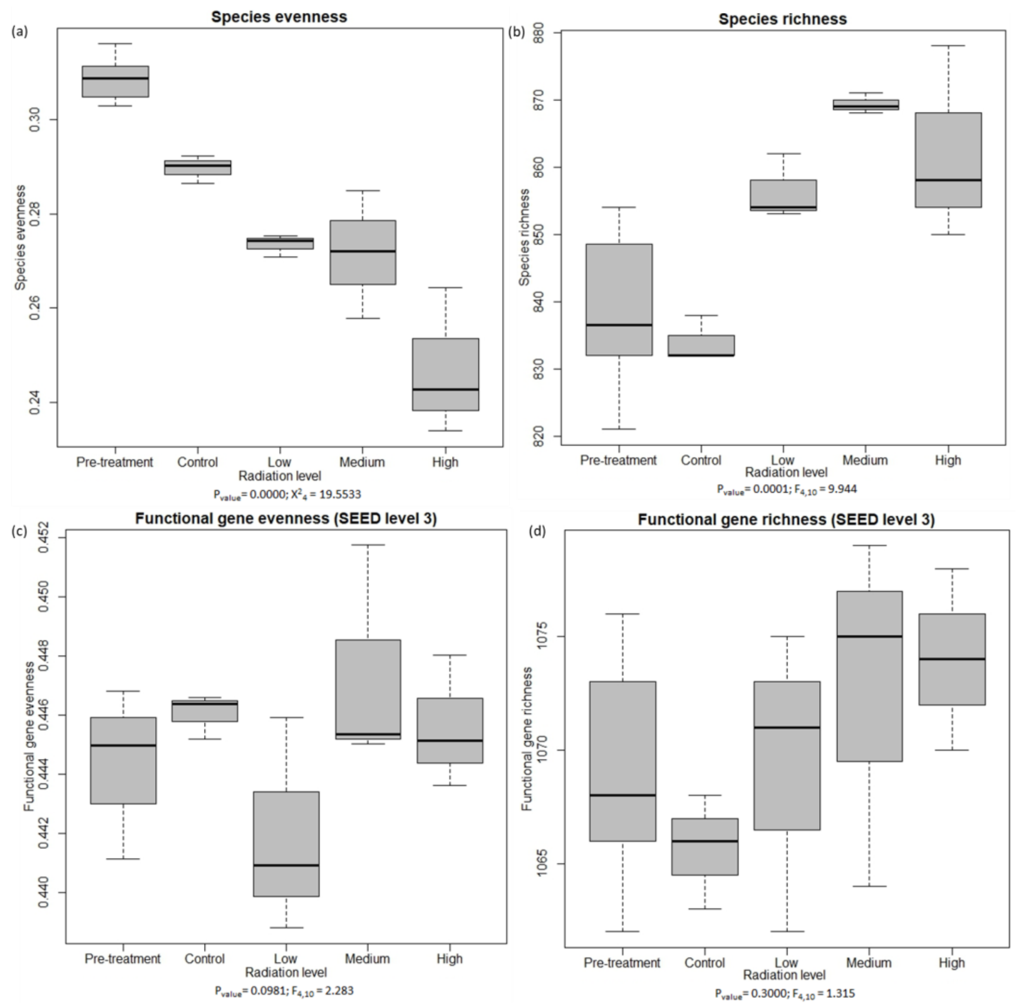

Supplementary Fig. 6. The species evenness and richness significantly reduced and increased with increasing radiation intensity respectively but the evenness of functional gene (SEED level 3) varied with treatment whereas the richness of functional genes (level 3) increased under irradiation.

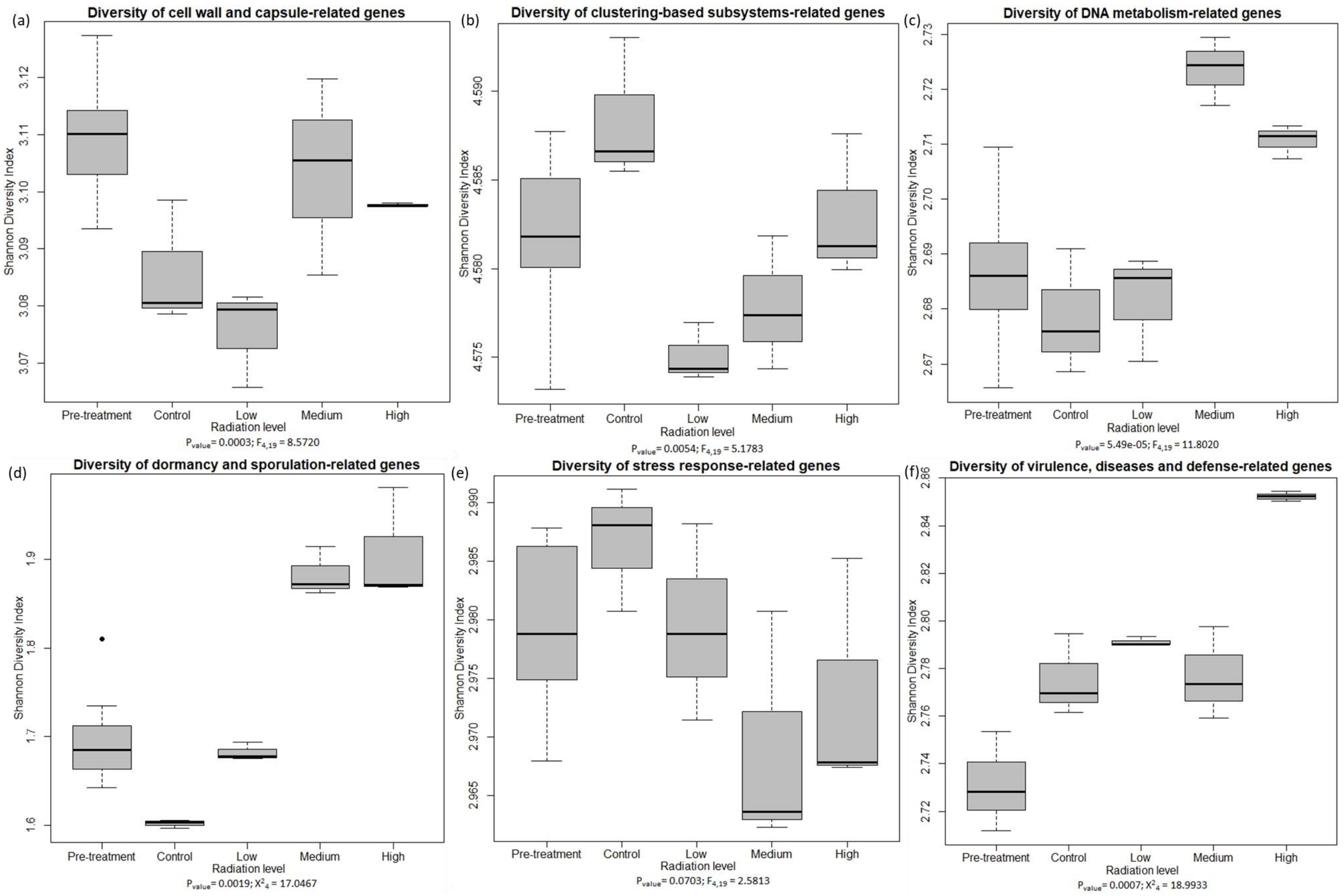
(a)
Diversity of cell wall and capsule-related genes
Shannon Diversity Index
Pre-treatment
Control
Low
Medium
High
Radiation level
P_{value}= 0.0003; $F_{4,19}$ = 8.5720
(b)
Diversity of clustering-based subsystems-related genes
Shannon Diversity Index
Pre-treatment
Control
Low
Medium
High
Radiation level
P_{value}= 0.0054; $F_{4,19}$ = 5.1783
(c)
Diversity of DNA metabolism-related genes
Shannon Diversity Index
Pre-treatment
Control
Low
Medium
High
Radiation level
P_{value}= 5.49e-05; $F_{4,19}$ = 11.8020
(d)
Diversity of dormancy and sporulation-related genes
Shannon Diversity Index
Pre-treatment
Control
Low
Medium
High
Radiation level
P_{value}= 0.0019; X^2_4 = 17.0467
(e)
Diversity of stress response-related genes
Shannon Diversity Index
Pre-treatment
Control
Low
Medium
High
Radiation level
P_{value}= 0.0703; $F_{4,19}$ = 2.5813
(f)
Diversity of virulence, diseases and defense-related genes
Shannon Diversity Index
Pre-treatment
Control
Low
Medium
High
Radiation level
P_{value}= 0.0007; X^2_4 = 18.9933

Supplementary Fig. 7. Shannon diversity index of SEED level 3 functional genes including cell wall and capsules (a), clustering based subsystems (b), DNA metabolism (c), dormancy and sporulation (d), stress response (e) and virulence, disease and defense-related genes.

Cell wall and capsule-related genes was most diverse in the pre-treatment samples closely followed by medium treated samples (a). In clustering based subsystems, Shannon diversity index was highest in the incubated samples (Control) with diversity increasing as radiation intensity increases (b). More so highest Shannon diversity index was obtained from medium, high radiation treated samples in DNA metabolism (c) and dormancy, and sporulation-related genes (d). In stress response-related genes, Shannon diversity index was lowest in medium and high radiation treated samples (e) but highest in the high radiation treated sample for virulence, disease and defence-related genes (f).

Supplementary Table 1. Sampling site details and descriptive statistics of shotgun metagenomics sequences

MG RAST ID	SAMPLE NAME	TREATMENT	TOTAL SEQUENCE	TOTAL BASEPAIRS	PREDICTED PROTEINS FEATURES	ANNOTATED PROTEIN (%)	TOTAL SEQUENCE AFTER QC	TOTAL BASEPAIRS AFTER QC
mgm4721502.3	0_C1	None	4,020,515	745,201,667	3,663,628	40.19	3,934,428	712,520,022
mgm4721506.3	0_C2	None	6,357,756	1,175,765,357	5,758,234	40.24	6,215,695	1,122,371,767
mgm4721490.3	0_C3	None	3,148,144	578,151,888	2,866,128	41.04	3,082,727	552,990,406
mgm4721496.3	0_L1	None	2,162,701	398,254,093	1,977,796	40.56	2,116,155	380,541,985
mgm4721494.3	0_L2	None	2,318,667	430,981,041	2,134,165	40.36	2,271,064	412,735,558
mgm4721497.3	0_L3	None	3,806,077	708,167,173	3,465,867	40.74	3,724,427	676,399,294
mgm4721492.3	0_M1	None	3,810,410	710,382,427	3,472,362	40.87	3,731,892	679,869,005
mgm4721488.3	0_M2	None	3,645,498	670,573,998	3,323,984	40.56	3,566,514	640,930,113
mgm4721498.3	0_M3	None	2,752,972	510,347,767	2,500,704	41.15	2,680,594	487,118,956
mgm4721486.3	0_H1	None	2,755,818	514,250,939	2,508,870	40.48	2,678,630	488,996,943
mgm4721504.3	0_H2	None	2,667,411	496,342,834	2,429,646	41.50	2,601,208	474,203,689
mgm4721487.3	0_H3	None	2,407,427	448,855,157	2,193,868	41.27	2,350,889	428,859,162
mgm4721505.3	6_C1	Incubated	3,155,188	589,106,649	2,872,552	42.30	3,082,270	561,024,270
mgm4721484.3	6_C2	Incubated	2,348,918	443,450,842	2,146,821	42.55	2,299,901	424,208,521
mgm4721500.3	6_C3	Incubated	2,498,942	458,426,991	2,271,650	42.10	2,442,713	436,928,719
mgm4721507.3	6_L1	Low	2,268,404	419,733,441	2,043,005	41.55	2,217,205	401,576,911
mgm4721485.3	6_L2	Low	2,410,004	444,400,399	2,171,479	41.61	2,352,304	424,580,126
mgm4721491.3	6_L3	Low	2,765,204	516,701,461	2,490,832	41.58	2,691,587	492,412,319
mgm4721503.3	6_M1	Medium	2,708,675	500,609,136	2,438,989	39.58	2,636,909	477,324,074
mgm4721495.3	6_M2	Medium	2,714,041	498,515,017	2,405,817	40.23	2,644,195	476,211,037
mgm4721489.3	6_M3	Medium	2,684,903	498,156,264	2,323,701	41.66	2,611,144	473,974,492
mgm4721493.3	6_H1	High	2,381,554	439,867,724	2,002,821	44.34	2,320,677	419,693,735
mgm4721499.3	6_H2	High	2,406,621	446,508,932	2,068,648	43.46	2,344,144	425,279,848
mgm4721501.3	6_H3	High	2,123,729	395,585,447	1,758,491	45.00	2,066,819	376,572,737

Key: Low (at 0.1 kGy), Medium (at (1 kGy) and High (at 3kGy) gamma ^{60}Co radiation treatment

Supplementary Table 2. The Eukaryota domain had 20 major phyla and 2 unclassified phyla. The unclassified unclassified (derived from Eukaryota) 1 phylum was present in the control samples whereas the unclassified unclassified (derived from Eukaryota) 2 was predominantly in the irradiated samples.

**Domain**	**Phyla**
Eukaryota	Apicomplexa
Eukaryota	Arthropoda
Eukaryota	Ascomycota
Eukaryota	Bacillariophyta
Eukaryota	Basidiomycota
Eukaryota	Blastocladiomycota
Eukaryota	Brachiopoda
Eukaryota	Chlorophyta
Eukaryota	Chordata
Eukaryota	Chytridiomycota
Eukaryota	Cnidaria
Eukaryota	Echinodermata
Eukaryota	Euglenida
Eukaryota	Hemichordata
Eukaryota	Microsporidia
Eukaryota	Mollusca
Eukaryota	Nematoda
Eukaryota	Phaeophyceae
Eukaryota	Platyhelminthes
Eukaryota	Streptophyta
Eukaryota	unclassified (derived from Eukaryota) 1
Eukaryota	unclassified (derived from Eukaryota) 2

Supplementary Table 3. Shannon index diversity for taxonomic group and SEED functional gene levels. Results showed all the different levels were statistically significant but for domain (at the sub system taxonomic level).

	**Taxonomic level**								**SEED Functional gene levels**			
	**Domain**	**Phylum**	**Order**	**Class**	**Family**	**Genus**	**Species**	**Strain**	**Level 1**	**Level 2**	**Level 3**	**Level 4**
X^2 or F value	14.313	19.173	16.889	16.875	19.02	19.553	19.153	19.153	16.107	7.053	18.463	17.328
P_{value}	0.006	0.000	0.002	0.002	0.000	0.000	0.000	0.000	0.002	0.001	2.43e-06	3.87e-06
DF	4	4	4	4	4	4	4	4,10	4	4,10	4,10	4,10
Control – Pre-treatment	+	+	*	*	+	*	*	*	*	+	+	+
High - Pre-treatment	+	*	+	+	*	*	*	*	+	+	*	*
Low - Pre-treatment	*	+	*	*	+	*	*	*	*	*	*	*
Medium - Pre-treatment	+	*	+	*	*	*	*	*	*	+	*	*
High – Control	+	*	*	*	*	*	*	*	*	+	*	*
Low – Control	+	+	+	+	+	+	+	+	*	+	*	+
Medium – Control	*	*	*	*	+	+	+	+	+	+	*	*
Low - High	+	*	*	*	*	*	*	*	*	*	*	*
Medium - High	*	+	+	+	*	*	*	*	+	*	+	+
Medium - Low	+	*	*	*	+	+	+	+	*	+	*	*

+ = Non-significant (at $P \leq 0.05$)

* = Significant (at $P \leq 0.05$)

Supplementary Table 4. Rank order nestedness of the samples, reordered in terms of species and functional genes. The results suggest that the radiation treated samples are nested within the control samples for both taxonomic species and functional gene categories.

Sample/Radiation level	Species Rank Order of Nestedness	Functional gene Rank Order of Nestedness
Pre-treatment 1	2	2
Pre-treatment 2	7	8
Pre-treatment 3	20	6
Pre-treatment 4	22	1
Pre-treatment 5	18	11
Pre-treatment 6	6	9
Pre-treatment 7	19	19
Pre-treatment 8	21	22
Pre-treatment 9	11	10
Pre-treatment 10	13	21
Pre-treatment 11	1	7
Pre-treatment 12	5	13
Control 1	10	17
Control 2	8	23
Control 3	16	12
Low 1	9	3
Low 2	24	5
Low 3	3	18
Medium 1	17	16
Medium 2	12	24
Medium 3	23	15
High 1	15	14
High 2	14	20
High 3	4	4
